# Routine HIV Screening in Portugal: Clinical Impact and Cost-Effectiveness

**DOI:** 10.1371/journal.pone.0084173

**Published:** 2013-12-18

**Authors:** Yazdan Yazdanpanah, Julian Perelman, Madeline A. DiLorenzo, Joana Alves, Henrique Barros, Céu Mateus, João Pereira, Kamal Mansinho, Marion Robine, Ji-Eun Park, Eric L. Ross, Elena Losina, Rochelle P. Walensky, Farzad Noubary, Kenneth A. Freedberg, A. David Paltiel

**Affiliations:** 1 Hôpital Bichat, Université Paris Diderot, Paris, France; 2 ATIP-Avenir Inserm: "Modélisation, Aide à la Décision, et Coût-Efficacité en Maladies Infectieuses,” Inserm U1137, Université Denis Diderot, Paris, France; 3 Escola Nacional de Saúde Pública, Universidade Nova de Lisboa, Lisbon, Portugal; 4 Instituto de Saúde Pública, Universidade do Porto, Porto, Portugal; 5 Centro Hospitalar de Lisboa Ocidental, Lisbon, Portugal; 6 Division of Infectious Diseases, Massachusetts General Hospital, Boston, Massachusetts, United States of America; 7 Harvard Medical School, Boston, Massachusetts, United States of America; 8 Division of General Medicine, Massachusetts General Hospital, Boston, Massachusetts, United States of America; 9 Medical Practice Evaluation Center, Massachusetts General Hospital, Boston, Massachusetts, United States of America; 10 Brigham and Women’s Hospital, Boston, Massachusetts, United States of America; 11 Harvard Center for AIDS Research, Boston, Massachusetts, United States of America; 12 Department of Epidemiology, Boston University School of Public Health, Boston, Massachusetts, United States of America; 13 Department of Biostatistics, Boston University School of Public Health, Boston, Massachusetts, United States of America; 14 The Institute for Clinical Research and Health Policy Studies, Tufts Medical Center, Boston, Massachusetts, United States of America; 15 Tufts Clinical and Translational Science Institute, Tufts University, Boston, Massachusetts, United States of America; 16 Department of Health Policy and Management, Yale School of Public Health, New Haven, Connecticut, United States of America; Alberta Provincial Laboratory for Public Health/ University of Alberta, Canada

## Abstract

**Objective:**

To compare the clinical outcomes and cost-effectiveness of routine HIV screening in Portugal to the current practice of targeted and on-demand screening.

**Design:**

We used Portuguese national clinical and economic data to conduct a model-based assessment.

**Methods:**

We compared current HIV detection practices to strategies of increasingly frequent routine HIV screening in Portuguese adults aged 18-69. We considered several subpopulations and geographic regions with varying levels of undetected HIV prevalence and incidence. Baseline inputs for the national case included undiagnosed HIV prevalence 0.16%, annual incidence 0.03%, mean population age 43 years, mean CD4 count at care initiation 292 cells/μL, 63% HIV test acceptance, 78% linkage to care, and HIV rapid test cost €6 under the proposed routine screening program. Outcomes included quality-adjusted survival, secondary HIV transmission, cost, and incremental cost-effectiveness.

**Results:**

One-time national HIV screening increased HIV-infected survival from 164.09 quality-adjusted life months (QALMs) to 166.83 QALMs compared to current practice and had an incremental cost-effectiveness ratio (ICER) of €28,000 per quality-adjusted life year (QALY). Screening more frequently in higher-risk groups was cost-effective: for example screening annually in men who have sex with men or screening every three years in regions with higher incidence and prevalence produced ICERs of €21,000/QALY and €34,000/QALY, respectively.

**Conclusions:**

One-time HIV screening in the Portuguese national population will increase survival and is cost-effective by international standards. More frequent screening in higher-risk regions and subpopulations is also justified. Given Portugal’s challenging economic priorities, we recommend prioritizing screening in higher-risk populations and geographic settings.

## Introduction

By comparison to its European neighbors, Portugal bears an unusually severe HIV/AIDS burden. Its overall HIV/AIDS prevalence is 0.53% [1,2]. Incidence estimates range from 0.005% to 1.08% per year, depending on risk group, and Portugal has the second highest incidence rate in the European Union [3]. Late presentation is common: 30.7% of newly detected individuals have already advanced to AIDS [1]. 

The Portuguese National Health Service provides universal coverage for HIV testing and care. To encourage earlier detection of infection and initiation of HIV care, the Portuguese parliament recently adopted a resolution to expand routine, population-based HIV testing [4]. This policy mirrors the recommendations of the World Health Organization (WHO), the United States Centers for Disease Control and Prevention, and the French Haute Autorité de Santé [5–7].

Portugal faces numerous challenges in implementing this resolution. Most notable among these is the current economic crisis. Portugal’s Gross Domestic Product (GDP) *per capita* (€15,600) is among the lowest in Western Europe; in 2010, debt service accounted for 33% of the overall National Health Service budget [8]. In the face of these challenges, we sought to estimate the survival benefits, costs, and cost-effectiveness of alternative approaches to expanded HIV screening in Portugal. 

## Methods

### Analytic Overview

We compared four strategies for HIV screening in Portuguese adults aged 18-69: 1) current practices of detection (notably non-routine, provider- or patient-initiated testing and presentation with opportunistic infections); versus current practices with the addition of 2) routine one-time screening; 3) routine screening every three years; and 4) routine annual screening. Given the geographic and demographic heterogeneity of the HIV/AIDS epidemic in Portugal, we also examined both regionally targeted screening and strategies focused on high-risk injection drug users (IDUs) and men who have sex with men (MSM). 

We used the Cost-Effectiveness of Preventing AIDS Complications (CEPAC) model, a widely-published microsimulation of HIV disease, to forecast mean costs, life-expectancy, and quality-adjusted life expectancy in months (QALM) in both HIV-infected and HIV-uninfected individuals under each strategy [9–17]. These estimates take into account the impact of the intervention on secondary HIV infection. 

Comparative value used the incremental cost-effectiveness ratios (ICERs), measured in both euros per year of life saved (€/YLS) and euros per quality-adjusted life-year saved (€/QALY). We applied the guidance of the WHO Commission on Macroeconomics and Health, which suggests that an intervention be labeled “cost-effective” in a given country if its ICER is less than three times the nation’s *per capita* GDP [18]. Since the Portuguese GDP is €15,600, programs delivering QALYs for less than €46,800 were deemed “cost-effective.” Because our goal was to inform decision making at the level of the Portuguese National Health Service, we conducted our analyses from a modified societal perspective (excluding indirect costs), and discounted costs, life expectancy, and quality adjusted life expectancy at 5% per annum [19].

### CEPAC Model

#### Disease Model

The Cost-Effectiveness of Preventing AIDS Complications (CEPAC) model presents the natural history, treatment, and costs of HIV disease [9–17]. The model portrays disease progression as a sequence of monthly transitions between “health states.” Each simulated patient is followed monthly in the model until death [9–17]. The progression of HIV disease is determined by CD4 count, HIV RNA level, and history of opportunistic infection. Patients receiving ART have reduced HIV RNA, increased CD4 count, and protection from opportunistic infection (OIs) [20]. Morbidity is calculated as a single outcome measure that expresses survival while accounting for quality of life [10,19,21,22]. Further details on the structure of the CEPAC model have been reported elsewhere [9–13]. The CEPAC model has been used to estimate the cost-effectiveness of expanded screening programs in the United States, South Africa, and France [12–15,17].

#### Screening Module

While the Disease Model described above simulates the course of HIV illness for all infected individuals, only patients with detected HIV infection who are successfully linked to care are eligible for clinic visits, OI prophylaxis, and antiretroviral therapy [12–15,17,23]. The Screening Module uses information on HIV prevalence, incidence, testing behavior, and HIV test performance to determine eligibility for treatment and conveys that information to the Disease Model. Persons in the Screening Module may either be HIV-uninfected or HIV-infected; the infected population is further separated into prevalent cases (persons infected at the time of model initialization [t=0]) and incident cases (whose infection occurs after model initialization). HIV detection takes place via one of three mechanisms: a) “background HIV testing,” defined as Portugal’s current non-routine testing practice; b) presentation to clinic with an AIDS-defining OI; or c) expanded, routine HIV screening. HIV is currently diagnosed in Portugal via one of the first two detection mechanisms. (Further details on the Screening Module are provided in Appendix S1.)

#### Transmission Module

The Transmission Module uses deterministic methods to link the effects of expanded HIV screening and earlier treatment initiation to both community HIV RNA levels and the subsequent number and timing of secondary HIV infections. The Transmission Module maintains a running tally of both the size of the susceptible (i.e. uninfected) population and the aggregate level of HIV RNA among infected (treated and untreated) persons. These are then combined with both Portuguese incidence data and HIV RNA-specific transmission rates from a variety of meta-analyses and modeling studies to estimate the number of new HIV transmissions in subsequent periods [1,24,25]. The Transmission Module permits us to estimate the impact of alternative HIV screening policies on transmissions from index cases to uninfected members of the population (i.e., first-order transmissions). By applying average discounted survival outcomes and costs from the CEPAC Disease Model to new infections, it also permits us to include the effects of secondary transmission in our cost-effectiveness estimates. (For further details on the Transmission Module, see Appendix S1.) 

### Input Data

Values for the key parameters used in our baseline assessment and plausible ranges we developed for the extensive sensitivity analyses are shown in Table 1. Further details on the protocols we used to assemble, develop, and validate these estimates are available in Appendix S1.

**Table 1:** Input Parameters for a Model of Routine HIV Screening in Portugal.

**Parameter**	**Baseline Value**	**Range for Sensitivity Analyses**	**Source**
**Cohort Characteristics**			
Mean age (SD), years, national population	43 (14)	38-48	[26]
Mean age (SD), years, MSM population	35 (12)	**--**	[27]
Mean age (SD), years, IDU population	31 (10)	**--**	[28]
Men, % of patients, national population	49	**--**	[26]
Men, % of patients, IDU population	71	**--**	[28]
**Efficacy of ART (% patients with HIV RNA suppression at 24 weeks)**			
1^st^ Line ART	86	73-99	[32]
2^nd^ Line ART	73	62-84	[37]
3^rd^ Line ART	61	52-70	[37]
4^th^ Line ART	65	55-75	[33]
5^th^ Line ART	40	34-46	[35,36]
6^th^ Line ART	15	13-17	[35]
**Treatment costs, 2012 €**			
1^st^ Line ART	780	550-1,014	[39]
2^nd^ Line ART	995	697-1,294	[39]
3^rd^ Line ART	1,072	750-1,394	[39]
4^th^ Line ART	1,569	1,098-2,040	[39]
5^th^ Line ART	2,259	1,581-2,937	[39]
6^th^ Line ART	888	622-1,154	[39]
**Prevalence of undiagnosed HIV, %**			
National population	0.16	0.03-0.30	[1,2]
IDUs	6.69	**--**	[2,54]
MSM	3.34	**--**	[2,55]
**Annual incidence rate, %**			
National population	0.03	0.01-0.04	[1]
IDUs	1.08	**--**	
MSM	0.43	**--**	
**Mean CD4 count (SD) at HIV care initiation in the “current practice” scenario, cells/μL**			
National population	292 (282)	**--**	[38]
IDUs	269 (260)	**--**	[38,56]
MSM	347 (336)	**--**	[38,56]
**Testing behavioral characteristics of proposed routine HIV screening program**			
Probability of test offer, %	80	--	Assumption
Probability of test acceptance, %	79	32-100*	[47]
Probability of linkage to care, %	78	20-100	[48]
**Costs associated with routine HIV screening program, 2012 €**			
HIV test cost	6	6-37	[49]
Confirmatory test (blood draw, Western Blot)	102	**--**	[49]
Post-test counseling costs for HIV+ patients	32	--	[49]
**Secondary transmission rate for heterosexuals and IDUs stratified by plasma HIV RNA (copies/mL),/100 person-years**			
> 50,000	9.0	3.9-21.1	[24]
10,000-49,999	8.1	2.8-23.8	[24]
3,500-9,999	4.2	0.8-20.7	[24]
400-3,499	2.1	0.6-7.5	[24]
<400	0.2	0.0-1.1	[24]
**Secondary transmission rate for MSMs stratified by plasma HIV RNA (copies/mL),/100 person-years**			
> 50,000	72.2	--	[24,25]
10,000-49,999	65.0	--	[24,25]
3,500-9,999	33.4	--	[24,25]
400-3,499	16.5	--	[24,25]
<400	1.6	--	[24,25]

SD: standard deviation; MSM: men who have sex with men; IDUs: injection drug users; ART: antiretroviral therapy

^*^ A sensitivity analysis was conducted on the product of *probability of test offer* and *probability of test acceptance*. We assigned a baseline value of 80% x 79% = 63% to this product and assumed a range of 32% to 100% for the sensitivity analysis.

#### Cohort Sociodemographic Characteristics

The baseline characteristics of the simulated cohort represent the Portuguese population aged 18 to 69 years old. The national population had a mean age of 43 years and was 49% male [26]. The MSM population had a mean age of 35 years (range 17-79 years) [27]. The IDU population had a mean age of 31 years (range 18-49). The IDU population was 71% male [28]. 

#### Disease Progression and Mortality

Recognizing the limited availability of Portuguese data on HIV natural history, we developed estimates using a combination of national and international sources and then subjected all estimates to extensive sensitivity analysis. Data from two French clinical cohorts were employed to estimate CD4-stratified OI incidence and HIV-related mortality rates [29]. National age- and gender-stratified mortality rates unrelated to HIV/AIDS were first obtained from Portuguese sources. These were further adjusted for MSM and IDUs using previously published age- and gender-stratified mortality ratios for these sub-populations [26,30]. 

#### Treatment

Simulated patients who were HIV diagnosed and effectively linked to care were assumed to receive CD4 count and HIV RNA measurements every four months [31]. In accordance with Portuguese national guidelines, patients who were HIV diagnosed and successfully linked to care received ART when their CD4 count was below 350 cells/μL or when they had a WHO Stage III or IV OI [31]. ART regimens were switched when patients were observed to have experienced virologic failure, defined as an HIV RNA rebound to pre-ART levels. Values for ART and OI prophylaxis efficacies were obtained from published randomized controlled trials (Table 1) [32–37]. Because these outcomes were derived from intention-to-treat analyses, we assumed that adherence to prophylaxis was comparable to the level of adherence observed in the clinical trials. The level of adherence to ART in clinical trials may be higher than in non-trial settings. As a result we may have overestimated ART effectiveness. However, lower efficacies, in particular for ART, were considered in the sensitivity analysis.

#### Costs and Quality of Life

Data on resource use for non-ART routine medical care and death were obtained from a survey of 5 Portuguese National Health System (NHS) hospitals [38]. ART regimen unit costs were the official prices paid by NHS hospitals and were obtained from the official Central Administration of the Health System (ACSS) procurement list [39]. Laboratory test unit costs were obtained from the Portuguese official tariffs. Unit costs for consultations were obtained from the Analytical Accounting of NHS Hospitals [40]. Quality of life values for given model events and health states were derived from published studies of health state utilities in HIV-infected patients [41,42]. All costs were converted to 2012 Euros [43].

#### HIV Prevalence and Incidence

Prevalence was derived using data from the National Institute for Health (INSA), the Portuguese agency responsible for completing compulsory data collection of HIV-positive diagnoses [1]. INSA reported a prevalence of 0.37% diagnosed cases in 2008. Applying a European report that 30% of all HIV cases are undiagnosed [2], we estimated a prevalence of undiagnosed HIV of 0.16% and an overall national prevalence of 0.53%. Prevalence data were obtained for twenty different country regions as well as for MSM and IDUs [1]. National and regional annual incidence rates for the national population and these sub populations were obtained from INSA and stratified by age by applying incidence rate ratios from a study of HIV incidence in the United States [1,44]. (The methods used to calculate risk group incidence are described in Appendix S1.)

#### Characteristics of “Background Testing”

In Portugal, HIV testing is performed on demand, and routinely proposed to pregnant women, patients with tuberculosis and sexually transmitted infections, and prisoners. Data from five large Portuguese hospitals, representing 38.1% of HIV patients in care in Portugal indicated that, for the year 2008, the mean CD4 count for all patients initiating HIV care with current testing practices was 292 cells/uL (SD 282) [38]. Mean CD4 at presentation to care for MSM (347 cells/μL (SD 336)) and IDUs (269 cells/μL (SD 260)) was derived by applying ratios from a French study that examined screening rates among the French national population, MSM and IDUs [17]. 

To determine the delay from infection to initiation of HIV care for the national population, we ran simulations where we offered no routine HIV screening and varied the non-routine screening rate. When we set the non-routine screening rate to once every 66 months, the mean CD4 count at presentation to care was 293 cells/μL, which closely approximates Portuguese surveillance data. This delay suggests a 1.52% constant monthly probability of background HIV testing, diagnosis and linkage to care. We repeated the same analysis for MSM and IDUs and determined that background testing occurs once every 39 months among MSM and once every 90 months among IDUs. 

To offer a conservative portrayal of the incremental benefits of expanded, routine HIV screening, we adopted a favorable view of background testing performance: 100% test sensitivity; 100% test specificity; and immediate linkage to care for 100% of detected cases. Doing so had the effect of understating the incremental benefits and cost-effectiveness of routine HIV testing, hence biasing the analysis against our own conclusions. Any retreat to a less favorable view of current practice would only serve to strengthen the findings reported below [45,46].

#### Characteristics of Routine Screening Program

For routine HIV screening in all settings and risk groups, we used a test offer rate of 80%, a test acceptance rate of 79%, and a linkage to care rate of 78% [47,48]. Testing was performed using a rapid HIV test without pre-test counseling, at a cost of €6 [49]. Patients with a positive result returned for a physician consult, a blood draw and confirmatory Western blot, with a total cost of €102 [49]. This physician visit included post-test counseling and linkage to care. We varied these parameters widely in sensitivity analyses for all three groups.

#### Secondary HIV Transmission

 For the national, regional and IDUs analyses, the estimated rate of HIV transmission ranged from 0.2/100 PY at HIV RNA levels <400 copies/mL to 9.0/100PY at HIV RNA levels >50,000 copies/mL. These values were derived from a meta-analysis of 11 studies that followed a total of 5,021 heterosexual couples with 461 observed HIV transmission events in eight countries [24]. For the MSM analysis, we assumed that transmission occurred at eight times the rate it does in the heterosexual population [25].

## Results

### Base Case – National Program

Under the “current practice” scenario, the discounted quality-adjusted life expectancy is 194.31 QALMs (undiscounted 464.86 QALMs) and the discounted per-person HIV-related costs are €630 for the national population (includes HIV-uninfected and HIV-infected people). In the HIV-infected population, the mean CD4 count at diagnosis is estimated at 293 cells/μL, and the discounted quality-adjusted life expectancy of HIV-infected persons is 164.09 QALMs (undiscounted 367.76 QALMs), (Table 2). 

**Table 2:** Base Case: Routine HIV Screening in Portuguese National Population.

**Variable**	**Current Practice**	**One-Time Screening**	**Screening Every Three Years**	**Annual Screening**
**General population**				
Mean undiscounted life expectancy, LM	465.14	465.19	465.24	465.29
Mean discounted life expectancy, LM	194.40	194.42	194.44	194.45
Mean undiscounted quality-adjusted life expectancy, QALM	464.86	464.90	464.96	465.00
Mean discounted quality-adjusted life expectancy, QALM	194.31	194.32	194.34	194.35
Mean discounted lifetime costs per person, €^1^	630	680	740	810
**HIV-infected population**				
Mean CD4 at detection, all cases (cells/µL)	293	306	353	398
Mean undiscounted life expectancy, LM^2^	407.84	414.29	422.78	428.76
Mean discounted life expectancy, LM^2^	177.98	181.05	183.25	185.44
Mean undiscounted quality-adjusted life expectancy, QALM^2^	367.76	373.36	381.42	387.14
Mean discounted quality-adjusted life expectancy, QALM^2^	164.09	166.83	168.93	170.96
Mean discounted lifetime costs per person, €^1^	91,410	97,610	103,740	109,680
Reduction in secondary cases over ten years	---	4.9%	5.3%	6.1%
QALM associated with transmission, discounted	70.00	70.01	70.02	70.02
Costs associated with transmission, discounted^1^	110	130	140	150
Incremental cost-effectiveness ratios, €/QALY^3^				
Without secondary transmission^4^	---	30,000	50,000	65,000
With secondary transmission^5^	---	28,000	44,000	59,000

^1^ Costs are rounded to the nearest €10.

^2^ Life-expectancy for HIV-infected represents the average life expectancy for all persons who have ever had HIV infection from the time of model entry.

^3^ ICERs are rounded to the nearest €1,000/QALY. The comparator strategy is always the next smallest, not dominated, alternative.

^4^ Incremental cost-effectiveness without secondary transmission = (cost per person in strategy 2 - cost per person in strategy 1)/ (QALMs per person in strategy 2 - QALMs per person in strategy 1)*12.

^5^ Incremental cost-effectiveness with secondary transmission = [(cost per person in strategy 2 + cost accrued per transmission in strategy 2) – (cost per person in strategy 1 + cost accrued per transmission in strategy 1)]/[(QALMs per person in strategy 2 + QALMs accrued per transmission in strategy 2) – (QALMs per person in strategy 1 + QALMs accrued per transmission in strategy 1)].

LM: life month; QALM: quality-adjusted life month; QALY: quality-adjusted life year; ICER: incremental cost-effectiveness ratio

After the addition of a one-time routine HIV screen to current practice, the quality-adjusted life expectancy is 194.32 QALMs (undiscounted 464.90 QALMs), and the average discounted lifetime HIV-related costs are €680/person, for the general population. The addition of a one-time routine HIV screen increases mean CD4 count at diagnosis to 306 cells/μL, and the quality-adjusted life expectancy to 166.83 QALMs (undiscounted 373.36 QALMs). The ICER of one-time screening compared to current practice is €30,000/QALY if one does not account for secondary transmission and €28,000/QALY if one does account for secondary transmission. This suggests that one-time screening is cost-effective (ICER less than €46,800, or three times Portuguese GDP). 

Screening patients every three years or annually further increases survival and costs. The ICERs for screening every three years are €50,000/QALY and €44,000/QALY, excluding and including secondary transmission, respectively. The ICER for annual screening is €65,000/QALY if secondary transmission is not considered and €59,000/QALY if it is considered. All further ICERs presented include the impact of secondary transmission.

### Regional Analysis

Compared to current practice, the addition of a one-time, routine HIV screening is cost-effective in each of the 20 different regions of Portugal (Table 3), with ICERs ranging from €27,000 to €38,000 per QALY. In six regions (Açores, Madeira, Porto, Faro, Setúbal, and Lisboa), increasing the screening frequency to every three years is also cost-effective, with ICERs ranging from €34,000 to €43,000 per QALY. Annual screening is not cost-effective in any of the regions.

**Table 3:** Results: Regional Analysis of Routine HIV Screening in Portugal.

**Region**	**Undiagnosed HIV Prevalence, %**	**Annual HIV Incidence, (/100 PY)**	**ICER (€/QALY)**		**ICER (€/QALY)**	
			**Without accounting for secondary transmission**		**Accounting for secondary transmission**	
			**One-Time Screening**	**Screening Every Three Years**	**One-Time Screening**	**Screening Every Three Years**
Guarda	0.03	0.005	40,000	97,000	36,000	96,000
Viana do Castelo	0.04	0.008	36,000	71,000	33,000	67,000
Vila Real	0.05	0.007	34,000	86,000	31,000	83,000
Viseu	0.05	0.009	34,000	71,000	33,000	91,000
Castelo Branco	0.05	0.011	31,000	66,000	29,000	61,000
Bragança	0.06	0.008	33,000	77,000	31,000	75,000
Braga	0.06	0.009	35,000	67,000	31,000	64,000
Aveiro	0.06	0.017	33,000	53,000	30,000	48,000
Açores	0.06	0.021	36,000	48,000	31,000	42,000
Portalegre	0.07	0.004	34,000	120,000	30,000	60,000
Évora	0.07	0.005	30,000	274,000	30,000	243,000
Coimbra	0.08	0.014	31,000	57,000	29,000	52,000
Beja	0.09	0.002	34,000	308,000	38,000	488,000
Leiria	0.09	0.015	34,000	56,000	31,000	50,000
Santarém	0.09	0.017	31,000	53,000	29,000	48,000
Madeira	0.09	0.023	33,000	47,000	30,000	41,000
Porto	0.19	0.031	30,000	45,000	28,000	39,000
Faro	0.21	0.035	29,000	41,000	27,000	35,000
Setúbal	0.26	0.030	32,000	41,000	30,000	36,000
Lisboa	0.29	0.046	28,000	40,000	27,000	34,000

1. ICERs are rounded to the nearest €1,000/QALY. The comparator strategy is always the next smallest, not dominated, alternative.

2. Dominated: less effective and more costly than some combination of alternative strategies, for the same region.

ICER: incremental cost-effectiveness ratio; QALY: quality-adjusted life year

### Risk Group Analysis

Among MSM, compared to current practice, not only one-time routine HIV-screening, but also annual screening is cost-effective (undiagnosed prevalence 3.34% and annual incidence 0.43%; ICER = €21,000/QALY). In IDUs, (undiagnosed prevalence 6.69%, annual incidence of 1.08%), all strategies are cost-effective, with ICERs ranging from €26,000 to €30,000 per QALY. Additional results from the risk group analysis are provided in Table 4. 

**Table 4:** HIV Screening in High Risk Groups in Portugal.

**Variable**	**Current Practice**	**One-Time Screening**	**Screening Every Three Years**	**Annual Screening**
**Men who have sex with men (undiagnosed HIV prevalence = 3.34%, annual HIV incidence = 0.43%)**				
**General population**				
Mean undiscounted life expectancy, LM	510.33	511.00	511.71	512.26
Mean discounted life expectancy, LM	202.94	203.28	203.45	203.67
Mean undiscounted quality-adjusted life expectancy, QALM	504.13	504.73	505.41	505.92
Mean discounted quality-adjusted life expectancy, QALM	200.82	201.12	201.28	201.49
Mean discounted lifetime costs per person, €^1^	14,830	15,500	16,060	16,650
**HIV-infected population**				
Mean undiscounted life expectancy, LM^2^	426.07	430.72	435.56	439.58
Mean discounted life expectancy, LM^2^	183.40	185.77	187.01	188.52
Mean undiscounted quality-adjusted life expectancy, QALM^2^	382.52	386.64	391.23	395.05
Mean discounted quality-adjusted life expectancy, QALM^2^	168.49	170.59	171.76	173.20
Mean discounted lifetime costs per person, €^1^	104,170	109,030	112,710	116,770
Percent reduction in secondary cases over ten years, %	---	3.21	3.44	4.41
QALM associated with transmission, discounted	794.42	795.55	796.01	796.66
Costs associated with transmission, discounted^2^	23,110	25,490	25,630	26,280
**Incremental cost-effectiveness ratios, €/QALY** ^3^				
Without secondary transmission^4^	---	26,000	Dominated^6^	37,000
With secondary transmission^5^	---	Dominated^6^	Dominated^6^	21,000
**Injection drug users (undiagnosed HIV prevalence = 6.69%, annual HIV incidence = 1.08%)**				
**General population**				
Mean undiscounted life expectancy, LM^2^	356.82	358.52	361.41	363.31
Mean discounted life expectancy, LM^2^	169.17	170.08	170.95	171.66
Mean undiscounted quality-adjusted life expectancy, QALM^2^	346.70	348.23	350.94	352.73
Mean discounted quality-adjusted life expectancy, QALM^2^	165.14	165.96	166.77	167.44
Mean discounted lifetime costs per person, €^1^	24,960	26,840	29,280	31,370
**HIV-infected population**				
Mean undiscounted life expectancy, LM^2^	324.55	330.21	339.86	346.27
Mean discounted life expectancy, LM^2^	160.00	163.07	165.98	168.35
Mean undiscounted quality-adjusted life expectancy, QALM^2^	290.69	295.74	304.80	310.85
Mean discounted quality-adjusted life expectancy, QALM^2^	146.49	149.26	151.99	154.23
Mean discounted lifetime costs per person, €^1^	83,730	89,910	98,030	104,960
Percent reduction in secondary cases over ten years	--	4.64	6.72	7.46
QALM associated with transmission, discounted	81.65	82.09	82.32	82.56
Costs associated with transmission, discounted^2^	4,470	5,360	5,380	5,560
**Incremental cost-effectiveness ratios, €/QALY** ^3^				
Without secondary transmission^4^	--	28,000	36,000	38,000
With secondary transmission^5^	--	26,000	28,000	30,000

^1^ Costs are rounded to the nearest €10.

^2^ Life-expectancy for HIV-infected represents the average life expectancy for all persons who have ever had HIV infection from the time of model entry.

^3^ ICERs are rounded to the nearest €1,000/QALY. The comparator strategy is always the next smallest, not dominated, alternative.

^4^ Incremental cost-effectiveness without secondary transmission = (cost per person in strategy 2 - cost per person in strategy 1)/ (QALMs per person in strategy 2 - QALMs per person in strategy 1)*12.

^5^ Incremental cost-effectiveness with secondary transmission = [(cost per person in strategy 2 + cost accrued per transmission in strategy 2) – (cost per person in strategy 1 + cost accrued per transmission in strategy 1)]/[(QALMs per person in strategy 2 + QALMs accrued per transmission in strategy 2) – (QALMs per person in strategy 1 + QALMs accrued per transmission in strategy 1)].

^6^ Dominated: higher cost-effectiveness ratio compared to preceding strategy.

LM: life month; QALM: quality-adjusted life month; QALY: quality-adjusted life year; ICER: incremental cost-effectiveness ratio

### Additional Sensitivity Analyses

**Figure 1:**
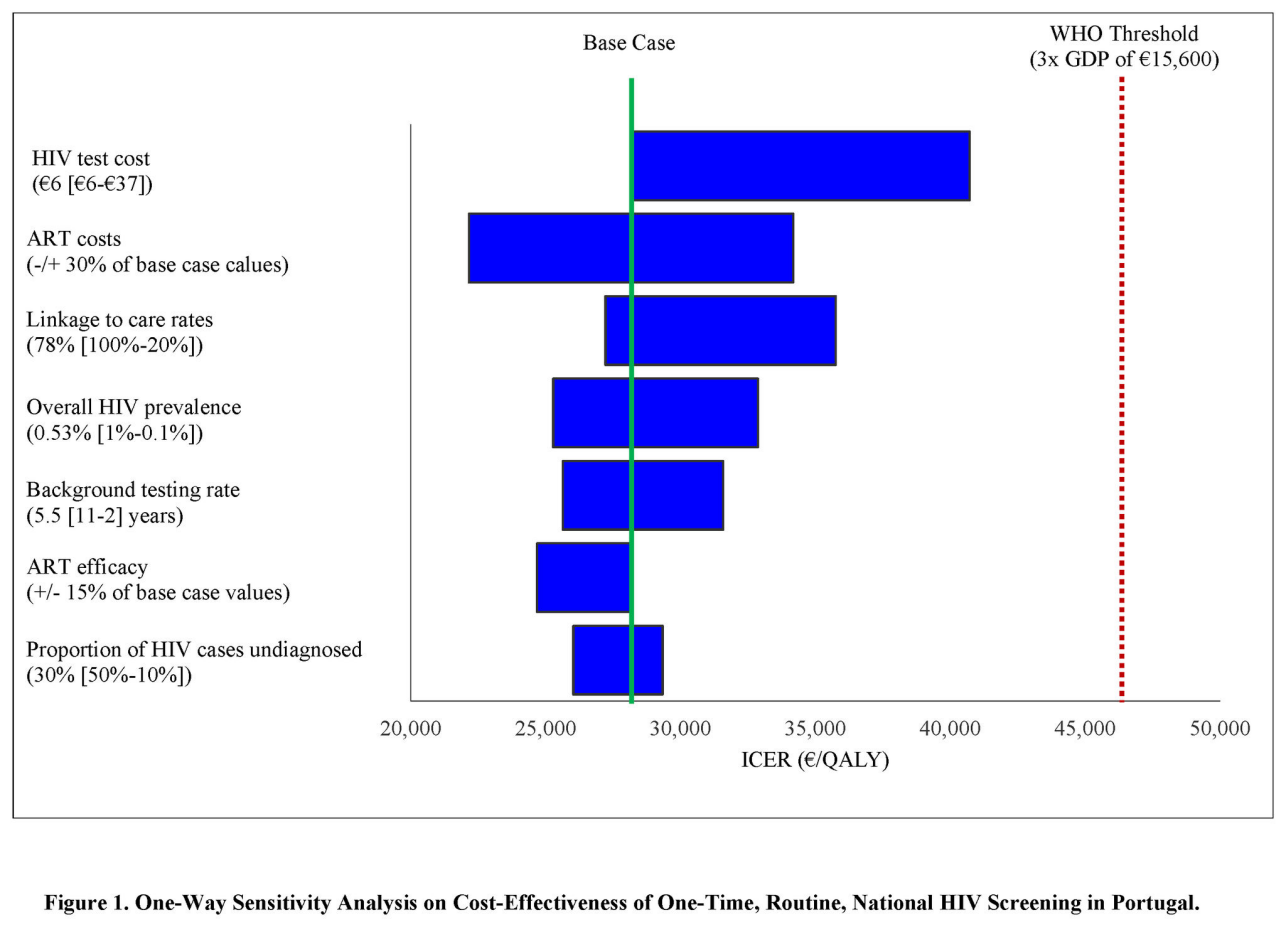
One-Way Sensitivity Analysis on Cost-Effectiveness of One-Time, Routine, National HIV Screening in Portugal. The width of the bar is the variation in the incremental cost-effectiveness ratio associated with alternative parameter values for that input, when secondary transmissions are taken into account. The numbers in parentheses next to each parameter on the y-axis indicate the base case value, and the numbers in brackets indicate the lower and upper-bounds used in the sensitivity analyses. QALY: quality-adjusted life year. ICER: incremental cost-effectiveness ratio.

In a series of one-way sensitivity analyses (Figure 1), we varied each of the key parameters across plausible ranges, while holding all other input values at baseline. The three parameters that caused the greatest change in the ICER for one-time screening were: linkage to care, the costs of ART, and HIV screening costs, but the ICER of one-time, routine, nationwide screening remained less than €46,800 (three times the Portuguese GDP of €15,600). (Additional one-way sensitivity analyses are reported in Appendix S1.) 

We then examined the effects of varying these most influential parameters simultaneously. At the baseline testing cost of €6, one-time screening is cost-effective whenever linkage to care exceeds 20%; this persists across the range of ART costs we considered. However, when testing costs are increased to €37 and ART costs are held at their baseline value, one-time routine screening is cost-effective only at linkage to care rates greater than 40%. At current ART and HIV test costs, one-time screening is cost-effective for HIV prevalence levels greater than 0.1% (undiagnosed HIV prevalence 0.03%). When the test cost is increased to €37, one time testing is cost-effective at an overall HIV prevalence of 0.3% (undiagnosed HIV prevalence 0.09%) or greater. (Further sensitivity analyses are reported in Appendix S1.)

### Impact of Expanded Screening on Secondary HIV Transmission

The addition of a national, one-time screen reduces the number of secondary transmissions by 4.9% over ten years. Screening every three years further reduces the number of secondary cases by 5.3%, while screening annually reduces the number of secondary cases over ten years by an additional 6.1% (see Appendix S1). When these HIV transmission effects are taken into account, screening every 3 years is cost-effective in six regions- including the four with the highest undiagnosed HIV prevalence and incidence (Porto, Faro, Setúbal and Lisboa, Table 3). 

## Discussion

One-time, routine, voluntary HIV screening in the Portuguese general population meets widely cited international standards of cost-effectiveness. More frequent screening among high-risk populations and in the four geographic regions with the highest HIV prevalence/incidence is also cost-effective. Among both MSM and IDUs, the returns to increased investment in screening are virtually proportional to incidence, suggesting that screening in these populations as often as yearly is warranted. Previous studies have demonstrated the cost-effectiveness of expanded testing in the specific context of middle-income countries (South Africa) and high-income countries (France, US) [12,13,17]. In the former case, cost-effectiveness was largely related to the low cost of ART and the very high burden of disease; in the latter case, results were driven in large measure by national income and the consequent willingness to pay for relatively small health gains. Portugal represents an intermediate case – a nation which, like many others, benefits from neither low ART prices nor very high GDP but which still bears the burden of a relatively high HIV prevalence and incidence. It is instructive, therefore, to find that a one-time, routine, voluntary HIV screening in the Portuguese general population still meets international standards of cost-effectiveness.

There is no universally accepted definition for a “cost-effective” ICER. The WHO offers general guidance and states that an ICER that is less than one time the per capita GDP for a specific country is “very cost-effective” in that context, and an ICER that is less than three times the per capita GDP is “cost-effective” [50]. These thresholds have been widely used to take into account national ability to pay. In Portugal, this sets a limit for an ICER to be considered cost-effective at $46,800. Anecdotal evidence suggests that the Portuguese National Authority of Medicines (Infarmed) adopts an informal threshold of €30,000/QALY in determining whether a given pharmaceutical intervention is cost-effective. This threshold is inspired by the United Kingdom National Institute for Health and Clinical Excellence (NIHCE) guidelines [51]. Our ICER estimate for a national one-time screening is just under that threshold. Thus, one-time, routine, voluntary HIV screening in the general population meets both widely cited international standards and informally understood standards of cost-effectiveness in Portugal. However, given the challenges of national implementation, as well as constrained resources, it may prove both practical and expedient to begin by initiating routine HIV screening first among IDUs and MSM and in the four high-prevalence regions, where the ICERs are unequivocally favorable, both by formal and informal standards.

At €28,000/QALY, the cost-effectiveness ratio for one-time, nation-wide screening in Portugal stands in contrast to higher ICERs, which we have previously obtained using similar methods and national data from both France (€60,400/QALY) and the United States (€67,400/QALY) [12,13,17]. These differences are explained by Portugal’s higher levels of undiagnosed HIV prevalence, higher HIV incidence, lower mean CD4 counts at presentation to care under current practices of HIV detection, and lower costs of HIV screening. Each of these differences leads to more favorable cost-effectiveness ratios for one-time routine screening in Portugal than in France or the United States [12,13,17]. 

While instructive to note the differences between this analysis and previous findings, it is also useful to highlight important qualitative similarities. First, we once again find that population-wide routine HIV screening is easily justified as a comparatively attractive use of scarce health resources. Second, we see that the cost-effectiveness of HIV screening is surprisingly insensitive to both the prevalence of undetected HIV in the target population and the cost of the HIV test. At current ART costs, only at undetected HIV prevalence levels less than or equal to 0.09% (overall prevalence of 0.3%) do the costs of counseling and testing itself begin to affect decision making [23,52]. Finally, we once again find that secondary transmission effects – while notable when measured on a population-wide basis over a long time horizon – exert minimal influence on the cost-effectiveness ratio [12]. 

This study has several limitations. First, the results are based on a mathematical model that estimates long-term consequences of different strategies based on data from multiple sources, some of which by necessity are not Portuguese. For example, the proportion of undiagnosed HIV cases (30%) is based on estimates for Europe and is not specific to Portugal [2]. Moreover, in the absence of available data in our regional population analysis, we assumed that the background testing rate is the same for all regions. The sensitivity analyses on these and other input parameters demonstrate that the results are robust to variations in these assumptions. However, while these sensitivity analyses were conducted to manage the uncertainties in our input data assumptions, a more complete exploration would have included a probabilistic sensitivity analysis (PSA), providing a cost-effectiveness acceptability curve showing the probability of cost-effectiveness of one-time screening against varying willingness-to-pay threshold values. Recognizing the difficulties of conducting PSA alongside a Monte Carlo microsimulation such as ours, we have adhered as closely as possible to the guidance of both the US Panel on Cost-effectiveness in Health and Medicine and the joint SMDM- ISPOR Task Force on Good Modeling Practice, both of which endorse the use of deterministic methods—as the one we have performed—to manage uncertainty [19,45,46]. Second, although we did account for the effect of HIV RNA levels on transmission, the population-level data we used for the heterosexual and IDU groups were based on a meta-analysis of studies of heterosexual, serodiscordant couples [24]. While we applied a multiplier to do this for our MSM analysis, this multiplier came from a model-based rather than a cohort study [25]. Furthermore, we only simulated first-order transmissions rather than use a fully dynamic HIV transmission model. Therefore, we may have under-represented the rates of infection and, therefore, the cost-effectiveness of HIV testing in high-risk populations. However, varying the probabilities of HIV transmission widely in sensitivity analyses produced no change in the cost-effectiveness of one-time routine screening. Finally, while we project that routine HIV testing will be cost-effective, this does not imply that it will necessarily be affordable; budget impact analysis will be a useful next step for budget planning [53].

In summary, we find that one-time routine HIV screening of the national population is a cost-effective intervention for Portugal. More frequent screening in higher-risk regions and subpopulations is also justified on both clinical and cost-effectiveness grounds. In light of Portugal’s current economic challenges, focusing on screening higher-risk populations and geographic settings should be a priority.

## Supporting Information

Appendix S1 **Technical Appendix.**
(DOCX)
