# Supplementary material for: Routine HIV Screening in Portugal: Clinical Impact and Cost-Effectiveness
Source: PLoS One. 2013 Dec 18;8(12):e84173. doi: 10.1371/journal.pone.0084173 (PMC3867470; doi:10.1371/journal.pone.0084173)
Supplement: Appendix S1 — Technical Appendix. (DOCX) [file pone.0084173.s001.docx]

**TECHNICAL APPENDIX**

**Routine HIV Screening in Portugal:**

**Clinical Impact and Cost-Effectiveness**

**Appendix S1**

Yazdan YAZDANPANAH, MD

Julian PERELMAN, PhD

Madeline A. DILORENZO, AB

Joana ALVES, MA

Henrique BARROS, MD

Céu MATEUS, PhD

João PEREIRA, PhD

Kamal MANSINHO, MD

Marion ROBINE, BS

Ji-Eun PARK, BS

Eric L. ROSS, BA

Elena LOSINA, PhD

Rochelle P. WALENSKY, MD, MPH

Farzad NOUBARY, PhD

Kenneth A. FREEDBERG, MD, MSc

A. David PALTIEL, PhD

**INTRODUCTION**

This analysis uses the Cost-Effectiveness of Preventing AIDS Complications (CEPAC) Model, a computer simulation of the clinical management and economics of HIV disease. The CEPAC Model comprises three distinct components: a Disease Model, which simulates the natural history of HIV illness and the impact of combination antiretroviral therapy on an infected individual; a Screening Module, which simulates the prevalence, incidence, detection, care and treatment of infected persons in a given population; and a Transmission Module, which links community HIV RNA levels to current numbers of infected and susceptible persons to predict the subsequent number and timing of secondary HIV infections. In this Technical Appendix, we describe in detail the mechanics of the Disease Model, the Screening Module and the Transmission Module. We explain the methods used to estimate undiagnosed HIV prevalence and incidence in several Portuguese sub-populations as well as the impact of expanded routine, voluntary HIV screening on HIV transmission. Finally, we present the results of additional sensitivity analyses conducted as part of this study.

**METHODS**

**CEPAC Model**

*Disease Model*

The Disease Model characterizes the natural history of illness in each individual patient as a sequence of monthly transitions from one “health state” to another. A random number generator and set of estimated probabilities determine how patients move between health states. Each patient’s clinical course – including all clinical events, the length of time spent in each health state, and the costs and quality-of-life associated with each health state – is tallied from entry into the model until death. Upon the patient’s death, his summary statistics are recorded. Cohort sizes are chosen so as to achieve statistical convergence for overall performance measures such as average life expectancy, quality-adjusted life expectancy, and lifetime medical costs. (This is typically achieved with sample sizes of 1 million patients.)

Health states describe the patient’s current health, relevant history, quality-of-life, and resource utilization patterns. They predict clinical prognosis, including disease progression, immune system deterioration, development and relapse of different opportunistic diseases, toxic reactions to medications, resistance to therapy, and mortality. The Disease Model defines three general categories of health states: chronic states, acute states, and death. Most of the time, patients reside in one of the chronic states, where progression of disease and immune system deterioration (CD4 decline) take place. Patients who develop an acute complication (e.g., an opportunistic disease or drug-related toxicity) temporarily move to an acute health state, where quality of life is lower and resource consumption levels and mortality rates are higher. Death can occur from either a particular opportunistic disease, chronic AIDS (e.g., wasting), or non-AIDS-related causes in either a chronic or an acute state.

Chronic and acute health states are stratified by: current and nadir CD4 cell count (>500 cells/μL; 301–500 cells/μL; 201–300 cells/μL; 101–200 cells/μL; 51–100 cells/μL; <50 cells/μL) and current and set-point HIV RNA level (>100,000 copies/mL; 30,001–100,000 copies/mL; 10,001–30,000 copies/mL; 3,001–10,000 copies/mL; 501–3,000 copies/mL; <500 copies/mL). Upon entry into the Disease Model, a patient is randomly assigned to a health state based upon a set of user-specified probability distributions. By permitting the user to define initial population distributions for patient age, sex, CD4 cell count, HIV RNA, and other demographic and clinical attributes, the model has the flexibility to explore a broad range of different patient cohorts.

At the start of each one-month cycle, the Disease Model records the patient’s CD4 cell count, HIV RNA level, history of acute illness, and current therapies to determine the probabilities that indicate movement to a new state in the subsequent month. Key parameters of the natural history of HIV disease, including rates of disease progression, opportunistic disease and mortality, as well as the effects of therapy, are derived from Portuguese and international clinical cohorts and public use datasets [[1](#_ENREF_1),[2](#_ENREF_2),[3](#_ENREF_3),[4](#_ENREF_4),[5](#_ENREF_5),[6](#_ENREF_6),[7](#_ENREF_7),[8](#_ENREF_8),[9](#_ENREF_9),[10](#_ENREF_10)]. HIV RNA levels determine the rate at which the CD4 counts decline. Patients with a history of opportunistic disease have a higher risk of recurrence, depending on CD4 count and current antiretroviral therapy (ART) use.

Patients can receive up to six sequential ART regimens. Patients who initially succeed on ART have a monthly probability of virologic failure and are considered to have failed a regimen after a repeated observed increase in HIV RNA level. The Disease Model accounts for a 12-month lag between virologic failure and CD4 decline.

In addition to life expectancy, quality-adjusted life expectancy, and total lifetime costs, model output may include disaggregated estimates of average cost per patient (e.g., drugs, laboratory tests, hospitalizations), performance of therapy (e.g., time on therapy, time to viral load rebound, rates of toxicity), and morbidity (specific opportunistic disease incidence rates, and causes of death).

*Screening Module*

The Screening Module simulates the epidemiology and detection of HIV infection in an at-risk population. It accounts for such parameters as HIV prevalence and incidence; HIV test sensitivity, specificity, and cost; screening frequency; and rates of test acceptance and linkage to care. This information is conveyed to the Disease Model, where it is linked to output on the course of untreated HIV disease and the timing of both AIDS-defining complications and non-AIDS-related mortality to establish whether, when, and how an individual case of HIV infection is first detected.

Figure S1 provides a conceptual overview of the Screening Module. Individual members of the population enter the simulation, one at a time. A random-number generator makes use of “time-to-event” probability distributions to assign each incoming person a unique set of four HIV-related event times: (1) time of HIV infection, t_i_; (2) time of next HIV test performed within the context of a specific routine HIV screening program, t_p_; (3) time of next non-routine HIV test based on the current HIV testing strategy ,t_b_; and (4) time of non-AIDS-related death, t_d._

User-specified HIV incidence can be stratified into seven age categories: <18 years, 18-25 years, 26-30 years, 31-35 years, 36-40 years, 41-45 years and >45 years. Incidence is modeled as a hazard rate and is assumed to be constant for each age category over time. Based on the monthly probability of infection, a random number generator determines the time to infection for each patient. Similarly, non-HIV-related mortality is determined by random number generation and probabilities based on standard life tables stratified by gender and age.

In the large majority of instances, t_i_ far exceeds t_d_, reflecting the reality that most people die uninfected. A simple “IF/THEN” statement (represented by the upper diamond in Figure S1) makes this determination; such cases will never enter the Disease Model. For some individuals, however, t_i_<t_d_ and HIV infection occurs during their lifetimes. At the given time t_i_, these patients proceed to the Disease Model, which transcribes their age at the moment of infection and begins simulating the progress of their illness and associated clinical and economic outcomes. These individuals are not eligible to receive any kind of HIV therapy within the Disease Model until and unless their HIV infection is identified. Thus, the Disease Model simulates disease progression for all infected individuals, but only detected cases are eligible for ART and opportunistic disease prophylaxis. Patients who are successfully detected and linked to care are assumed to receive services (and incur associated costs) that conform to Portuguese national guidelines. These include CD4 count and HIV RNA laboratory monitoring every four months, as well as *Pneumocystis jiroveci* pneumonia prophylaxis when CD4 count falls below 200 cells/μL, *Mycobacterium avium* complex prophylaxis when CD4 count falls below 50 cells/μL, toxoplasmosis prophylaxis when CD4 count falls below 100 cells/μL and ART when CD4 count falls below 350 cells/μL or when one acquires an opportunistic infection [[11](#_ENREF_11),[12](#_ENREF_12)].

Detection of HIV infection can occur via one of three discrete mechanisms. First, an infected individual can receive an HIV-positive test result within the context of a specific routine screening program (t_b_>t_p_>t_i_). Second, an infected individual can receive an HIV-positive test result within the context of a non-routine testing mechanism corresponding here to the Portuguese current testing practice (t_p_>t_b_>t_i_). Third, an infected individual can be detected after seeking medical care for an AIDS-defining illness. The Screening Module determines the time of detection via the first two mechanisms, using a random number generator to combine user-specified assumptions regarding HIV test accuracy, rates of test acceptance, return for results, linkage-to-care, and non-routine testing, as obtained from national Portuguese data (Table S1). Program costs are also determined. This information is conveyed to the Disease Model which, in turn, determines the actual time of detection by comparing the time of screen-detection to the time of the first OI.

The Screening Module defines four distinct HIV states: HIV-negative, HIV-acute, asymptomatic HIV-chronic, and symptomatic HIV-chronic. Newly infected patients transition to the HIV-acute state for one month before entering the chronic, HIV-asymptomatic state [[13](#_ENREF_13)].

Each individual is tracked from the time of entry into the Screening Module until death or transition to the Disease Model. Upon each patient’s departure from the Screening Module, summary statistics are recorded and a new patient simulation begins. Sample size requirements depend upon user-specified prevalence, incidence, and testing program performance. Outputs of the Screening Module include: total program enrollment by HIV status; test acceptance and return rates by HIV status; frequency of testing and total number and type of tests performed; total true- and false-positive tests and true- and false-negative tests; CD4 count and HIV RNA at detection; time from infection to diagnosis; mechanism of detection; and total testing-related costs.

*CEPAC Transmission Module*

The purpose of the CEPAC transmission module is to incorporate the effects of HIV transmission into overall cost-effectiveness calculations. In a closed cohort cost-effectiveness analysis, the incremental cost-effectiveness ratio (ICER) of a treatment strategy is calculated based on the lifetime healthcare costs and survival under two different strategies, as shown in Equation 1:

| $ICER=\frac{C_{2}-C_{1}}{Y_{2}-Y_{1}}$ | (1) |
| --- | --- |

Here, **C_1_** denotes the total HIV-related healthcare cost of the cohort accrued under strategy 1 (e.g. one-time testing), **Y_1_** is the total survival (in quality-adjusted person-years) of the cohort under strategy 1, and **C_2_** and **Y_2_** are the respective outcomes under strategy 2 (e.g. yearly testing).

To include the impact of transmissions from this initial closed cohort, we amend this formula as shown in Equation 2:

| $ICER=\frac{\left( C_{2}+\mathrm{Cs}_{2} \right)-\left( C_{1}+\mathrm{Cs}_{1} \right)}{\left( Y_{2}+\mathrm{Ys}_{2} \right)-\left( Y_{1}+\mathrm{Ys}_{1} \right)}$ | (2) |
| --- | --- |

Here **Cs_2_** indicates the cost associated with second-order transmitted HIV cases under strategy 2, **Ys_2_** is the survival associated with second-order transmitted HIV cases under strategy 2, and **Cs_1_** and **Ys_1_** are the respective outcomes under strategy 1.

For an analysis of HIV screening policies, any hypothetical strategy will impact both HIV-infected and uninfected individuals; screening of negative individuals will increase costs, and any quality of life reductions associated with false-positive tests will harm the negative population. For this reason, all of the terms in the above equation (including the secondary transmission terms) must include both infected and uninfected individuals.

The purpose of the Transmission Module is to calculate the values of the terms **Cs_i_** and **Ys_i_** from Equation 2. This process takes place in three steps:

1. Projecting the number and timing of transmissions under a given strategy: An average baseline transmission rate per infected person is calculated in each month **m** of the analysis. This is based on two things: 1) a user-specified transmission rate for individuals in each of seven viral load categories ( **r(v)** ), expressed in terms of transmissions per 100 PY; and 2) the number of infected individuals in each viral load category in each month ( **N_inf_(m,v)** ), based on the results of the disease model simulation. The following formula is used to calculate the baseline transmission rate:

| $r_{base}(m)=\frac{\sum_{v=1}^{7} N_{inf}(m,v)*r(v)}{\sum_{v=1}^{7} N_{inf}(m,v)}$ | (3) |
| --- | --- |

To calculate the absolute number of transmissions in month **m**, this baseline transmission rate is then multiplied by 1) the total number of infected individuals alive in month **m** (**N_inf_(m)** ); and 2) the age-adjusted relative sexual activity level of the cohort in month **m** ( **a(m)** ), as shown below:

| $T(m)=r_{base}(m)*N_{inf}(m)*a(m)$ | (4) |
| --- | --- |

1. Back-calculating the size of the susceptible pool: The transmission calculations described in the previous section make no assumptions regarding the existence, or specific characteristics, of any pool of susceptible individuals. However, as described above, any attempt to incorporate transmissions into cost-effectiveness results must account for the negative individuals in the “secondary cohort.” We use published estimates of the HIV incidence rate to back-calculate the size of this negative pool. The method relies on the premise that the number of transmissions at any time-point is equal to the incidence rate times the size of the susceptible pool at that time-point. We use the standard-of-care (SOC) strategy to calculate the baseline size of this susceptible pool; any strategy that increases transmissions compared to SOC will remove individuals from this pool; any strategy that decreases transmissions will result in more individuals staying in the susceptible pool.

Mechanistically, the transmission module first calculates an average transmission rate for the SOC strategy over a lifetime horizon, using the following formula:

| $r_{avg}={\sum_{m=0}^{\infty} T(m)}/{\sum_{m=0}^{\infty} N_{inf}(m)}$ | (5) |
| --- | --- |

With this average transmission rate, a “smoothed” number of transmissions in any month can then be calculated as:

| $T_{smoothed}(m)=r_{avg}(m)*N_{inf}(m)$ | (6) |
| --- | --- |

The difference between **T(m)** and **T_smoothed_(m)** lies in their sensitivity to viral load variation: while **T(m)** varies with changes in the cohort’s viral load, **T_smoothed_(m)** is insensitive to month-to-month variation in the cohort’s viral load distribution caused by treatment initiation or failure. Since the size of the susceptible pool should not vary with these month-to-month changes in viral load, we use **T_smoothed_(m)** to calculate the size of the susceptible pool; this is done using the following formula:

| ${Ns}_{neg}(m)= \frac{T_{smoothed}(m)}{I}$ | (7) |
| --- | --- |

where **I** is the HIV incidence rate in the region of interest, and **Ns_neg_(m)** is the size of the negative pool in the secondary cohort at month **m**.

1. Projecting cost and survival: After the number of transmissions in each month is calculated, the Transmission Module projects the survival of all individuals, with and without HIV, in the secondary cohort. First, the CEPAC disease model is used to determine average survival trajectories for HIV-infected and uninfected individuals. Then the Transmission Module must determine how many new infections have occurred each month, and how many uninfected individuals have remained uninfected in each month.

To do this, the Transmission Module first adds **T(m)** infected survival trajectories each month, discounted to the time at which these transmissions occurred. Next, the module compares the values of **T(m)** under the strategy of interest to those from the SOC. If the strategy of interest prevents **n** transmissions at month **m**, then **n** uninfected survival trajectories will be added to susceptible pool, starting at month **m**. Conversely, if the strategy of interest results in **n** additional transmissions, then **n** uninfected survival trajectories must be removed from the susceptible pool. Finally, the total number of discounted life-years accrued by both the infected and uninfected populations is summed to give **Ys_i_**, the total survival of the secondary cohort.

An analogous method is used to project the costs accrued by the secondary cohort. Cost trajectories, derived from the results of CEPAC disease model simulations, are applied to all individuals in the secondary cohort. The total costs, from both the infected and uninfected populations, are then summed to give **Cs_i_**, the total costs accrued by the secondary cohort.

**Input Data**

Only specific inputs that were not explained in the manuscript are described here.

*Costs and Quality of Life*

Data on the direct costs of routine medical care were obtained from a survey conducted at five NHS hospitals during 2008 [[14](#_ENREF_14)]. This survey collected exhaustive information on health resource use, including consultations, in-patient stays, lab tests and drugs. Patients were included in the survey if their primary diagnosis was HIV/AIDS or an OI related to HIV/AIDS. Given the low number of deaths in the survey, monthly death costs were calculated using deceased patients’ hospitalization costs, using data from all in-patient discharges at NHS hospitals for the year 2007, provided by the Central Administration of the Health System (ACSS) [[15](#_ENREF_15)]. If the in-patient length of stay was below 30 days, the value was adjusted by using patient questionnaire-derived daily costs for patients with AIDS-defining events. ART unit costs were defined as the official prices paid by NHS hospitals and were obtained from the official ACSS procurement list [[16](#_ENREF_16)]. Laboratory test unit costs were obtained from the Portuguese official tariffs [[17](#_ENREF_17)]. Unit costs for consultations were obtained from the 2006 Analytical Accounting of NHS Hospitals [[15](#_ENREF_15)]. Quality of life values for given model events and health states were derived from published studies of health state utilities in HIV-infected patients [[18](#_ENREF_18),[19](#_ENREF_19)]. All model costs were converted to 2012 euros by applying GDP deflators from the International Monetary Fund World Economic Outlook database [[20](#_ENREF_20)].

*HIV Prevalence and Incidence*

Prevalence was calculated using data from the National Institute for Health (INSA). By 2008, there were a total of 25,904 reported diagnosed alive cases among the Portuguese population aged 18-69 years out of a total population of 7,068,134 people aged 18-69 years [[21](#_ENREF_21)]. This yielded a national diagnosed HIV prevalence of 0.37%. Based on a well-known study of diagnosed versus undiagnosed HIV prevalence in Europe, we assumed that 70% of cases were diagnosed, and derived an overall national HIV prevalence of 0.53%, as shown below [[22](#_ENREF_22)]:

70% * (Overall HIV Prevalence) = Diagnosed HIV Prevalence

70% * (Overall HIV Prevalence) = 0.37%

Overall HIV Prevalence = 0.37%/70%

Overall HIV Prevalence = 0.53%

Therefore, the undiagnosed HIV prevalence used in the model was the diagnosed HIV prevalence subtracted from the overall HIV prevalence, or 0.53%-0.37% = 0.16%. The undiagnosed HIV prevalence was however varied in the sensitivity analysis. Prevalence data was also obtained for twenty different country regions, MSM and IDU. National and regional annual incidence rates were obtained from INSA (Table S1). Annual incidence rates for each risk group were calculated by first conducting a model simulation to estimate the average undiscounted life expectancy of an HIV-infected individual in that risk group who receives ART from the time of diagnosis. Then, the risk group’s HIV prevalence was divided by this life expectancy in order to determine the risk group’s HIV incidence rate. All incidence was stratified by age by applying incidence rate ratios from a study of HIV incidence in the United States [[23](#_ENREF_23)].

*Characteristics of Background Testing*

Data from five large Portuguese hospitals representing 38.1% of HIV patients under care in Portugal (University Hospital of Coimbra, São João Hospital from Porto and Hospital Centre of Setúbal) indicated that for the year 2008 the mean CD4 count for all patients initiating HIV care under current screening practices was 292 cells/uL (SD 282 cells/μL) [[14](#_ENREF_14)]. This same value was used for all regional populations since CD4 data for each region examined was not available.

Since there were no clinical data available on the mean CD4 at presentation to care for MSM and IDU, we referred to a French study that examined screening rates among the French national population, MSM and IDU [[24](#_ENREF_24)]. In the French study, the mean CD4 at HIV presentation to care was 372 cells/μL (SD 257 cells/μL) for the national population, 442 cells/μL (SD 289 cells/μL) for MSM and 342 cells/μL (SD 180 cells/μL) for IDU. Therefore, the ratio of the French national population CD4 count at presentation to care to the MSM CD4 count at presentation to care is 0.84, and the ratio of the general population CD4 at presentation to care to the IDU CD4 at presentation to care is 1.09. Applying these ratios to the Portuguese national population mean CD4 at presentation to care yields a mean CD4 at presentation to care of 347 cells/μL for MSM and 269 cells/μL for IDU.

In order to determine the delay from infection to initiation of HIV care for the national population, we ran a number of simulations where we varied only the non-routine HIV screening rate and offered no routine HIV screening. When we set the non-routine screening rate to once every 66 months, the mean CD4 count at presentation to care was 293 cells/μL, which closely approximates Portuguese clinical data. This delay suggests a 1.52% constant monthly probability of non-routine HIV screening, diagnosis and linkage to care. We therefore assumed that non-routine screening occurred once every 66 months, under conditions of perfect test offer rates, perfect test acceptance rates and perfect linkage to care rates. We repeated the same analysis for MSM and IDU and determined that background testing occurs once every 39 months (2.56% monthly probability) among MSM and once every 90 months among IDU (1.11% monthly probability).

For the national population, we conducted sensitivity analyses where we examined a broad range of background testing rates ranging from once every 12 months (8.33% monthly probability) to once every 156 months (0.64% monthly probability).

**RESULTS**

Results reported in the main manuscript provide detailed information regarding input parameters and sensitivity analyses on the most influential input parameters. Herein, we provide in more detail input variables and results of further sensitivity analyses on other parameters of interest (Tables S2-S4).**REFERENCES**

1. Gallant JE, DeJesus E, Arribas JR, Pozniak AL, Gazzard B, et al. (2006) Tenofovir DF, emtricitabine, and efavirenz vs. zidovudine, lamivudine, and efavirenz for HIV. N Engl J Med 354: 251-260.

2. Grinsztejn B, Nguyen B, Katlama C, Gatell J, Lazzarin A, et al. (2007) Safety and efficacy of the HIV-1 integrase inhibitor raltegravir (MK-0518) in treatment-experienced patients with multidrug-resistant virus: a phase II randomised controlled trial. Lancet 369: 1261-1269.

3. Havlir DV, Dube MP, Sattler FR, Forthal DN, Kemper CA, et al. (1996) Prophylaxis against disseminated Mycobacterium avium complex with weekly azithromycin, daily rifabutin, or both. N Engl J Med 335: 392-398.

4. Ioannidis JPA, Cappelleri JC, Skolnik PR, Lau J, Sacks HS (1996) A meta-analysis of the relative efficacy and toxicity of pneumocystis carinii prophylactic regimens. Arch Intern Med 156: 177-188.

5. Johnson M, Grinsztejn B, Rodriguez C, Coco J, DeJesus E, et al. (2005) Atazanavir plus ritonavir or saquinavir, and lopinavir/ritonavir in patients experiencing multiple virological failures. AIDS 19: 685-694.

6. Lalezari JP, Goodrich J, deJesus E, Lampiris H, Gulick R, et al. (2007) Efficacy and safety of maraviroc plus optimized background therapy in viremic ART-experienced patients infected with CCR5-tropic HIV-1: 24-week results of a phase 2b/3 study in the US and Canada [abstract 104bLB]. Presented at the 14th Conference on Retroviruses and Opportunistic Infections, Los Angeles, CA.

7. Lidman C, Berglund O, Tynell E, Lindback S, Elvin K (1994) Aerosolized pentamidine as primary prophylaxis for Pneumocystis carinii pneumonia: efficacy, mortality and morbidity. AIDS 8: 935-939.

8. Mellors JW, Munoz A, Giorgi JV, Margolick JB, Tassoni CJ, et al. (1997) Plasma viral load and CD4+ lymphocytes as prognostic markers of HIV-1 infection. Ann Intern Med 126: 946-954.

9. Nelson M, Arasteh K, Clotet B, Cooper DA, Henry K, et al. (2005) Durable efficacy of enfuvirtide over 48 weeks in heavily treatment-experienced HIV-1-infected patients in the T-20 versus optimized background regimen only 1 and 2 clinical trials. J Acquir Immune Defic Syndr 40: 404-412.

10. Yazdanpanah Y, Chene G, Losina E, Goldie SJ, Merchadou LD, et al. (2001) Incidence of primary opportunistic infections in two human immunodeficiency virus-infected French clinical cohorts. Int J Epidemiol 30: 864-871.

11. Coordenação Nacional para a infecção de VIH/SIDA (2009) Recomendações portuguesas para o tratamento da infecção VIH-SIDA. Available: <http://www.aidsportugal.com/Modules/WebC_Docs/GetDocument.aspx?DocumentId=82>. Accessed 20 July 2013.

12. Centers for Disease Control and Prevention (2009) Guidelines for prevention and treatment of opportunistic infections in HIV-infectd adults and adolescents. Recommendations from CDC, the National Institutes of Health, and the HIV Medicine Association of the Infectious Diseases Society of America. Morbidity and Mortality Weekly Report. Available: <http://www.cdc.gov/mmwr/pdf/rr/rr5804.pdf>. Accessed 17 March 2013.

13. Bartlett JG, Gallant JE (2001) 2001-2002 Medical management of HIV infection. Baltimore: Johns Hopkins University, Division of Infectious Diseases.

14. Perelman J, Alves J, Miranda A, Mateus C, Mansinho K, et al. (2013) Direct treatment costs for HIV/AIDS in Portugal. Rev Saúde Pública, In press.

15. Administração Central do Sistema de Saúde (2007) Contabilidade analitica 2006 hospitais do SNS. Available: <http://www.acss.min-saude.pt/Portals/0/DownloadsPublicacoes/SNS/Info_gestao/Contab_Analitica_2006_Hospitais_SNS.pdf>. Accessed 17 June 2013.

16. Ministerio da Saúde (2013) Catálogo do aprovisionamento público da saúde. Available: <http://www.catalogo.min-saude.pt/caps/publico/default.asp>. Accessed 22 March 2013.

17. Ministério da Saúde (2009) Portaria nº 132/2009. Diário da República, 1.ª série - N.º 21.

18. Paltiel AD, Scharfstein JA, Seage GR, Losina E, Goldie SJ, et al. (1998) A Monte Carlo simulation of advanced HIV disease: application to prevention of CMV infection. Med Decis Making 18: S93-S105.

19. Schackman BR, Goldie SJ, Freedberg KA, Losina E, Brazier J, et al. (2002) Comparison of health state utilities using community and patient preference weights derived from a survey of patients with HIV/AIDS. Medical Decision Making 22: 27-38.

20. International monetary fund (2013) World Economic Outlook Database. Available: <http://www.imf.org/external/pubs/ft/weo/2004/01/data/index.htm>. Accessed 23 April 2013.

21. Departamento de doenças infecciosas, unidade de referência e vigilância epidemiológica, núcleo de vigilância laboratorial de doenças nfecciosas (2012) Programa Nacional para a Infeção VIH/SIDA. Infeção VIH/SIDA: A Situação em Portugal a 31 de Dezembro de 2011. Lisbon: Instituto Nacional de Saúde

22. Hamers F, Phillips A (2008) Diagnosed and undiagnosed HIV-infected populations in Europe. HIV Med 9: 6-12.

23. Prejean J, Song R, Hernandez A, Ziebell R, Green T, et al. (2011) Estimated HIV Incidence in the United States, 2006–2009. PLoS ONE 6: e17502.

24. Yazdanpanah Y, Sloan CE, Charlois-Ou C, Le Vu S, Semaille C, et al. (2010) Routine HIV screening in France: clinical impact and cost-effectiveness. PLoS ONE 5: e13132.

25. Portuguese National Institute of Statistics (2011) Portuguese statistical data for 2009-2011. Available: <http://www.ine.pt/xportal/xmain?xpid=INE&xpgid=ine_base_dados>. Accessed 16 June 2013.

26. Frange P, Meyer L, Deveau C, Tran L, Goujard C, et al. (2012) Recent HIV-1 infection contributes to the viral diffusion over the French territory with a recent increasing frequency. PLoS ONE 7: e31695.

27. Esteves A, Parreira R, Piedade J, Venenno T, Franco M, et al. (2003) Spreading of HIV-1 subtype G and envB/gagG recombinant strains among injecting drug users in Lisbon, Portugal. AIDS Res Hum Retroviruses 19: 511-517.

28. Mathers BM, Degenhardt L, Phillips B, Wiessing L, Hickman M, et al. (2008) Global epidemiology of injecting drug use and HIV among people who inject drugs: a systematic review. Lancet 372: 1733-1745.

29. The European MSM internet survey (EMIS) community report 1.eu. Available: <http://www.emis-project.eu/sites/default/files/public/publications/EMIS_2010_CommunityReport1_English_0.pdf>. Accessed 12 May 2013.

30. Jauffret-Raustide M, Couturier E, Le Strat Y, Barin F, Emmanuelli J, et al. (2006) Assessment of HIV and HCV seroprevalence and drug-users profiles, InVS-ANRS Coquelicot Study, France, 2004. Bulletin Epidemiologique Hebdomadaire 33: 244-247.

31. Rodrigues J, Marques P, Pimentel L (2011) Testes reativos CAD VIH/SIDA Porto. Poster session presented at VIH Portugal, Lisbon, Portugal.

32. Attia S, Egger M, Muller M, Zwahlen M, Low N (2009) Sexual transmission of HIV according to viral load and antiretroviral therapy: systematic review and meta-analysis. AIDS 23: 1397-1404.

33. McCormick AW, Walensky RP, Lipsitch M, Losina E, Hsu H, et al. (2007) The effect of antiretroviral therapy on secondary transmission of HIV among men who have sex with men. Clin Infect Dis 44: 1115-1122.

| **Table S1. Input parameters for a model of routine HIV screening in Portugal** | | | | | | | | | | | | |
| --- | --- | --- | --- | --- | --- | --- | --- | --- | --- | --- | --- | --- |
| **Parameter** | | | | | **Baseline Value** | | | | **Range** | | **Source** | |
| **Cohort Characteristics** | | | | | | | | | | | | |
| *Mean age (SD), years* | | | | | | | | | | | | |
| National population | | | | | | 43 (14) | | | | 38-48 | | [[25](#_ENREF_25)] |
| MSM population | | | | | | 35 (12) | | | | **--** | | [[26](#_ENREF_26)] |
| IDU population | | | | | | 31 (10) | | | | **--** | | [[27](#_ENREF_27)] |
| *Male sex, % of patients* | | | | | | | | | | | | |
| National population | | | | | | 49 | | | | **--** | | [[25](#_ENREF_25)] |
| IDU population | | | | | | 71 | | | | **--** | | [[27](#_ENREF_27)] |
| **Disease Progression** | | | | | | | | | | | | |
| *Mean monthly decline in CD4 count by HIV RNA level (copies/mL), cells/μL (SD)* | | | | | | | | | | | | |
| >30,000 | | | | | | 6.4 (0.3) | | | | **--** | | [[8](#_ENREF_8)] |
| 10,001 – 30,000 | | | | | | 5.4 (0.2) | | | | **--** | | [[8](#_ENREF_8)] |
| 3,001 – 10,000 | | | | | | 4.6 (0.2) | | | | **--** | | [[8](#_ENREF_8)] |
| 501 – 3,000 | | | | | | 3.7 (0.2) | | | | **--** | | [[8](#_ENREF_8)] |
| <500 | | | | | | 3.0 (0.3) | | | | **--** | | [[8](#_ENREF_8)] |
| *Monthly probability of opportunistic disease by CD4 count (cells/μL), %* | | | | | | | | | | | | |
|  | <50 | 51-100 | 101-200 | 201-300 | | | 301-500 | >500 | |  | |  |
| PCP | 1.17 | 0.35 | 0.35 | 0.17 | | | 0.06 | 0.06 | | **--** | | [[10](#_ENREF_10)] |
| MAC | 0.52 | 0.30 | 0.16 | 0.02 | | | 0.02 | 0.00 | | **--** | | [[10](#_ENREF_10)] |
| TOXO | 0.66 | 0.14 | 0.25 | 0.13 | | | 0.11 | 0.03 | | **--** | | [[10](#_ENREF_10)] |
| CMV | 0.87 | 0.36 | 0.16 | 0.02 | | | 0.09 | 0.02 | | **--** | | [[10](#_ENREF_10)] |
|  |  |  |  |  | | |  |  | |  | |  |
| **Table S1. (continued)** | | | | | | | | | | | | |
| **Parameter** | | | | | | **Baseline Value** | | | | **Range** | | **Source** |
| FUNG | 0.94 | 0.22 | 0.22 | 0.07 | | | 0.09 | 0.04 | | **--** | | [[10](#_ENREF_10)] |
| OTHER | 2.37 | 1.53 | 0.91 | 0.69 | | | 0.34 | 0.20 | | **--** | | [[10](#_ENREF_10)] |
| *Mortality attributable to acute opportunistic disease, %* | | | | | | | | | | | | |
| PCP | | | | | | 7.0 | | | | **--** | | [[10](#_ENREF_10)] |
| MAC | | | | | | 16.9 | | | | **--** | | [[10](#_ENREF_10)] |
| TOXO | | | | | | 9.5 | | | | **--** | | [[10](#_ENREF_10)] |
| CMV | | | | | | 9.0 | | | | **--** | | [[10](#_ENREF_10)] |
| FUNG | | | | | | 0.0 | | | | **--** | | [[10](#_ENREF_10)] |
| **Treatment** | | | | | | | | | | | | |
| *ART efficacy at 48 weeks, % HIV RNA <400 copies/mL (mean increase in CD4 count, cells/μL)* | | | | | | | | | | | | |
| 1^st^ line ART | | | | | | 81 (190) | | | | -- | | [[1](#_ENREF_1)] |
| 2^nd^ line ART | | | | | | 70 (110) | | | | -- | | [[5](#_ENREF_5)] |
| 3^rd^ Line ART | | | | | | 58 (121) | | | | -- | | [[5](#_ENREF_5)] |
| 4^th^ Line ART | | | | | | 65* (102)* | | | | -- | | [[2](#_ENREF_2)] |
| 5^th^ Line ART | | | | | | 40* (121) | | | | -- | | [[6](#_ENREF_6),[9](#_ENREF_9)] |
| 6^th^ Line ART | | | | | | 12 (45) | | | | -- | | [[9](#_ENREF_9)] |
| **Costs and quality of life** | | | | | | | | | | | | |
| *ART costs, 2012 €* | | | | | | | | | | | | |
| 1^st^ line ART | | | | | | 780 | | | | 550-1,014 | | [[16](#_ENREF_16)] |
|  | | | | | |  | | | |  | |  |
| 2^nd^ line ART | | | | | | 995 | | | | 697-1,294 | | [[16](#_ENREF_16)] |
|  | | | | | |  | | | |  | |  |
| **Table S1. (continued)** | | | | | | | | | | | | |
| **Parameter** | | | | | | **Baseline Value** | | | | **Range** | | **Source** |
| 3^rd^ Line ART | | | | | | 1,072 | | | | 750-1,394 | | [[16](#_ENREF_16)] |
| 4^th^ Line ART | | | | | | 1,569 | | | | 1,098-2,040 | | [[16](#_ENREF_16)] |
| 5^th^ Line ART | | | | | | 2,259 | | | | 1,581-2,937 | | [[16](#_ENREF_16)] |
| 6^th^ Line ART | | | | | | 888 | | | | 622-1,154 | | [[16](#_ENREF_16)] |
| *Laboratory test costs, 2012 €* | | | | | | | | | | | | |
| CD4 test | | | | | | 37 | | | | -- | | [[17](#_ENREF_17)] |
| HVL test | | | | | | 53 | | | | -- | | [[17](#_ENREF_17)] |
| *Consultation costs, 2012 €* | | | | | | | | | | | | |
| Physician consult | | | | | | 32 | | | | -- | | [[15](#_ENREF_15)] |
| *Health-related quality of life by CD4 count (cells/μl), %* | | | | | | | | | | | | |
| ≤50 | | | | | | 83 | | | | -- | | [[19](#_ENREF_19)] |
| 51-200 | | | | | | 85 | | | | -- | | [[19](#_ENREF_19)] |
| 201-500 | | | | | | 86 | | | | -- | | [[19](#_ENREF_19)] |
| >500 | | | | | | 87 | | | | -- | | [[19](#_ENREF_19)] |
| *Health-related quality of life by opportunistic disease, %* | | | | | | | | | | | | |
| PCP | | | | | | 73 | | | | -- | | [[18](#_ENREF_18)] |
| MAC | | | | | | 69 | | | | -- | | [[18](#_ENREF_18)] |
| TOXO | | | | | | 69 | | | | -- | | [[18](#_ENREF_18)] |
| CMV | | | | | | 78 | | | | -- | | [[18](#_ENREF_18)] |
| FUNG | | | | | | 78 | | | | -- | | [[18](#_ENREF_18)] |
| OTHER | | | | | | 69 | | | | -- | | [[18](#_ENREF_18)] |
|  | | | | | |  | | | |  | |  |
|  | | | | | | | | | | | | |
| **Table S1. (continued)** | | | | | | | | | | | | |
| **Parameter** | | | | | | **Baseline Value** | | | | **Range** | | **Source** |
| **HIV Prevalence and Incidence** | | | | | | | | | | | | |
| *Prevalence of undiagnosed HIV, %* | | | | | | | | | | | | |
| National population | | | | | | 0.16 | | | | 0.03-0.30 | | [[21](#_ENREF_21),[22](#_ENREF_22)] |
| IDU | | | | | | 6.69 | | | | **--** | | [[22](#_ENREF_22),[28](#_ENREF_28)] |
| MSM | | | | | | 3.34 | | | | **--** | | [[22](#_ENREF_22),[29](#_ENREF_29)] |
| *Annual incidence rate, %* | | | | | | | | | | | | |
| National population | | | | | 0.03 | | | | | 0.01-0.04 | | [[21](#_ENREF_21)] |
| IDU | | | | | 1.08 | | | | | **--** | |  |
| MSM | | | | | 0.43 | | | | | **--** | |  |
| **Characteristics of Background Testing** | | | | | | | | | | | | |
| *Mean CD4 (SD) at care initiation, cells/μL* | | | | | | | | | | | | |
| National population | | | | | 292 (282) | | | | | _ | | [[14](#_ENREF_14)] |
| IDU | | | | | 269 (260) | | | | | -- | | [[14](#_ENREF_14),[24](#_ENREF_24)] |
| MSM | | | | | 347 (336) | | | | | **--** | | [[14](#_ENREF_14),[24](#_ENREF_24)] |
| *Non-routine screening rate, monthly probability, %* | | | | | | | | | | | | |
| National population | | | | | 1.52 | | | | | 0.64-8.33 | | Calculated |
| IDU | | | | | 1.11 | | | | | **--** | | Calculated |
| MSM | | | | | 2.56 | | | | | **--** | | Calculated |
|  | | | | |  | | | | |  | |  |
|  | | | | |  | | | | |  | |  |
|  | | | | |  | | | | |  | |  |
| **Table S1. (continued)** | | | | | | | | | | | | |
| **Parameter** | | | | | **Baseline Value** | | | | | **Range** | | **Source** |
| **Characteristics of Routine Screening Program** | | | | | | | | | | | | |
| *Behavioral characteristics, %* | | | | |  | | | | |  | |  |
| Probability of test offer, % | | | | | 80 | | | | | -- | | Assumption |
| Probability of test acceptance, % | | | | | 79 | | | | | 32-100** | | [[30](#_ENREF_30)] |
| Probability of linkage to care, % | | | | | 78 | | | | | 20-100 | | [[31](#_ENREF_31)] |
| *Testing costs, 2012 €* | | | | |  | | | | |  | |  |
| HIV rapid test | | | | | 6 | | | | | 6-37 | | [[17](#_ENREF_17)] |
| Confirmatory test (blood draw, Western Blot) | | | | | 102 | | | | | **--** | | [[17](#_ENREF_17)] |
| Post-test counseling for HIV+ patients | | | | | 32 | | | | | -- | | [[17](#_ENREF_17)] |
| **Secondary HIV Transmission Rate According to Plasma Viral Load (copies/mL), 100 person-years** | | | | | | | | | | | | |
| *National population and IDU* | | | | | | | | | | | | |
| > 50,000 | | | | | 9.0 | | | | | 3.9-21.1 | | [[32](#_ENREF_32)] |
| 10,000-49,999 | | | | | 8.1 | | | | | 2.8-23.8 | | [[32](#_ENREF_32)] |
| 3,500-9,999 | | | | | 4.2 | | | | | 0.8-20.7 | | [[32](#_ENREF_32)] |
| 400-3,499 | | | | | 2.1 | | | | | 0.6-7.5 | | [[32](#_ENREF_32)] |
| <400 | | | | | 0.2 | | | | | 0.0-1.1 | | [[32](#_ENREF_32)] |
| *MSM* | | | | |  | | | | |  | |  |
| > 50,000 | | | | | 72.2 | | | | | **--** | | [[32](#_ENREF_32),[33](#_ENREF_33)] |
| 10,000-49,999 | | | | | 65.0 | | | | | **--** | | [[32](#_ENREF_32),[33](#_ENREF_33)] |
| 3,500-9,999 | | | | | 33.4 | | | | | **--** | | [[32](#_ENREF_32),[33](#_ENREF_33)] |
| 400-3,499 | | | | | 16.5 | | | | | **--** | | [[32](#_ENREF_32),[33](#_ENREF_33)] |
| <400 | | | | | 1.6 | | | | | **--** | | [[32](#_ENREF_32),[33](#_ENREF_33)] |

PCP: *Pneumocystisjiroveci* pneumonia; MAC: *Mycobacterium avium* complex; TOXO: toxoplasmosis; CMV: cytomegalovirus; FUNG: fungal infections; OTHER: other OIs

* In the absence of data at 48 weeks, we used the mean increase in CD4 count for 4^th^ line at 24 weeks and we present the treatment efficacy for 4^th^ and 5^th^ line at 24 weeks here.

** A sensitivity analysis was conducted on the product of *probability of test offer* and *probability of test acceptance*. We assigned a baseline value of 80% x 79% = 63% to this product and assumed a range of 32% to 100% for the sensitivity analysis.

**Table S2. Selected univariate sensitivity analyses: one-time, national, routine screening**

| **Testing strategy** | **Costs (€)^1,2^** | | **QALMs, total population^1^** | **Transmissions per HIV-infected person** | **QALMs accrued per transmission^2^** | **Costs accrued per transmission^1^** | **ICER (€/QALY)^3^** |
| --- | --- | --- | --- | --- | --- | --- | --- |
| *Base Case Analysis* | | | | | | | |
| Current practice | 630 | 194.31 | | 0.60 | 70.00 | 110 | -- |
| One-time screening | 680 | 194.32 | | 0.60 | 70.01 | 130 | 28,000 |
| *30% reduction in cost of all lines of ART* | | | | | | | |
| Current practice | 480 | 194.31 | | 0.60 | 70.00 | 90 | -- |
| One-time screening | 520 | 194.32 | | 0.60 | 70.01 | 100 | 22,000 |
| *30% increase in cost of all lines of ART* | | | | | | | |
| Current practice | 770 | 194.31 | | 0.60 | 70.00 | 140 | -- |
| One-time screening | 830 | 194.32 | | 0.60 | 70.01 | 160 | 34,000 |
| *HIV test cost with inclusion of pre-test counseling, €37 (Base case = €6)* | | | | | | | |
| Current practice | 627 | 194.31 | | 0.60 | 69.91 | 111 | -- |
| One-time screening | 697 | 194.32 | | 0.61 | 69.92 | 134 | 41,000 |

**Table S2. (continued)**

| **Testing strategy** | | **Costs (€)^1,2^** | | **QALMs, total population^1^** | | **Transmissions per HIV-infected person** | | **QALMs accrued per transmission^2^** | | **Costs accrued per transmission^1^** | | **ICER (€/QALY)^3^** | |
| --- | --- | --- | --- | --- | --- | --- | --- | --- | --- | --- | --- | --- | --- |
| *Overall HIV prevalence = 0.1% (Base case = 0.53%)* | | | | | | | | | | | | | |
| Current practice | | 460 | | 194.43 | | 0.60 | | 51.37 | | 80 | | -- | |
| One-time screening | | 470 | | 194.43 | | 0.60 | | 51.38 | | 100 | | 33,000 | |
| *Overall HIV prevalence = 1.0% (Base case = 0.53%)* | | | | | | | | | | | | | |
| Current practice | | 820 | | 194.18 | | 0.59 | | 90.51 | | 140 | | -- | |
| One-time screening | | 900 | | 194.21 | | 0.61 | | 90.52 | | 170 | | 25,000 | |
| *Background testing rate = every 2 years (Base case rate = every 5.5 years)* | | | | | | | | | | | | | |
| Current practice | | 710 | | 194.34 | | 0.61 | | 71.03 | | 130 | | -- | |
| One-time screening | | 740 | | 194.35 | | 0.61 | | 71.03 | | 130 | | 32,000 | |
| *Background testing rate = every 11 years (Base case rate = every 5.5 years)* | | | | | | | | | | | | | |
| Current practice | | 580 | | 194.28 | | 0.59 | | 69.67 | | 100 | | -- | |
| One-time screening | | 630 | | 194.31 | | 0.60 | | 69.68 | | 130 | | 26,000 | |

**Table S2. (continued)**

| **Testing strategy** | **Costs (€)^1,2^** | | **QALMs, total population^1^** | **Transmissions per HIV-infected person** | **QALMs accrued per transmission^2^** | **Costs accrued per transmission^1^** | **ICER (€/QALY)^3^** |
| --- | --- | --- | --- | --- | --- | --- | --- |
| *Proportion of HIV cases undiagnosed = 10% (Base case = 30%)* | | | | | | | |
| Current practice | 490 | 194.40 | | 0.60 | 54.71 | 90 | -- |
| One-time screening | 510 | 194.41 | | 0.60 | 54.72 | 100 | 29,000 |
| *Proportion of HIV cases undiagnosed = 50% (Base case = 30%)* | | | | | | | |
| Current practice | 770 | 194.21 | | 0.59 | 85.33 | 140 | -- |
| One-time screening | 840 | 194.24 | | 0.61 | 85.35 | 160 | 26,000 |
| *Linkage to care = 20% (Base Case = 78.4%)* | | | | | | | |
| Current practice | 630 | 194.30 | | 0.60 | 70.02 | 110 | -- |
| One-time screening | 640 | 194.31 | | 0.60 | 70.02 | 120 | 36,000 |
| *Linkage to care = 100% (Base Case = 78.4%)* | | | | | | | |
| Current practice | 630 | 194.30 | | 0.60 | 69.93 | 110 | -- |
| One-time screening | 690 | 194.33 | | 0.61 | 69.95 | 130 | 27,000 |

**Table S2. (continued)**

| **Testing strategy** | | **Costs (€)^1,2^** | | **QALMs, total population^1^** | | **Transmissions per HIV-infected person** | | **QALMs accrued per transmission^2^** | | **Costs accrued per transmission^1^** | | **ICER (€/QALY)^3^** | |
| --- | --- | --- | --- | --- | --- | --- | --- | --- | --- | --- | --- | --- | --- |
| *HIV test acceptance = 32% (Base Case = 63%)* | | | | | | | | | | | | | |
| Current practice | | 630 | | 194.31 | | 0.60 | | 69.99 | | 110 | | -- | |
| One-time screening | | 650 | | 194.32 | | 0.60 | | 70.00 | | 120 | | 29,000 | |
| *HIV test acceptance = 100% (Base Case = 63%)* | | | | | | | | | | | | | |
| Current practice | | 630 | | 194.30 | | 0.60 | | 70.04 | | 110 | | -- | |
| One-time screening | | 700 | | 194.33 | | 0.61 | | 70.05 | | 140 | | 28,000 | |
| *Lowest estimates for Attia transmission coefficients* | | | | | | | | | | | | | |
| Current practice | | 630 | | 194.31 | | 0.23 | | 26.96 | | 40 | | -- | |
| One-time screening | | 680 | | 194.32 | | 0.23 | | 26.96 | | 50 | | 29,000 | |
| *Highest estimates for Attia transmission coefficients* | | | | | | | | | | | | | |
| Current practice | | 630 | | 194.31 | | 1.64 | | 192.78 | | 310 | | -- | |
| One-time screening | | 680 | | 194.32 | | 1.67 | | 192.80 | | 360 | | 28,000 | |

**Table S2. (continued)**

| **Testing strategy** | | **Costs (€)^1,2^** | | **QALMs, total population^1^** | | **Transmissions per HIV-infected person** | | **QALMs accrued per transmission^2^** | | **Costs accrued per transmission^1^** | | **ICER (€/QALY)^3^** | |
| --- | --- | --- | --- | --- | --- | --- | --- | --- | --- | --- | --- | --- | --- |
| *ART efficacy reduced by 15% at 24 weeks, % HIV RNA <400 copies/mL (mean increase in CD4 count, cells/μL): 73%/62%/52%/55%/34%/13% for 1^st^, 2^nd^, 3^rd^, 4^th^, 5^th^ and 6^th^ line ART* | | | | | | | | | | | | | |
| Current practice | | 590 | | 194.28 | | 0.59 | | 71.72 | | 110 | | -- | |
| One-time screening | | 640 | | 194.30 | | 0.60 | | 71.73 | | 130 | | 28,000 | |
| *ART efficacy increased by 15% at 24 weeks, % HIV RNA <400 copies/mL (mean increase in CD4 count, cells/μL): 99%/84%/70%/75%/46%/17% for 1^st^, 2^nd^, 3^rd^, 4^th^, 5^th^ and 6^th^ line ART* | | | | | | | | | | | | | |
| Current practice | | 660 | | 194.33 | | 0.59 | | 67.98 | | 110 | | -- | |
| One-time screening | | 700 | | 194.35 | | 0.60 | | 67.99 | | 120 | | 25,000 | |

1. Costs are rounded to the nearest €10 and discounted at 5% per annum.
2. QALMs are discounted at 5% per annum.
3. Incremental cost-effectiveness with secondary transmission = [(cost per person in strategy 2 + cost accrued per transmission in strategy 2) – (cost per person in strategy 1 + cost accrued per transmission in strategy 1)]/[(QALMs per person in strategy 2 + QALMs accrued per transmission in strategy 2) – (QALMs per person in strategy 1 + QALMs accrued per transmission in strategy 1)]. ICERs are rounded to the nearest €1,000/QALY.

**Table S3. Multi-way sensitivity analysis impact of linkage to care, HIV testing costs and ART costs on one-time, national, routine screening**

| \| \| \| \| **Testing strategy** \| **Costs (€)^1,2^** \| \| **QALMs, total population^1^** \| \| **Transmissions per HIV-infected person** \| \| **QALMs accrued per transmission^2^** \| \| **Costs accrued per transmission^1^** \| \| **ICER (€/QALY)^3^** \| \| \| --- \| --- \| --- \| --- \| --- \| --- \| --- \| --- \| --- \| --- \| --- \| --- \| --- \| \| ***Linkage to care = 20%*** \| \| \| \| \| \| \| \| \| \| \| \| \| \| *30% decrease in cost of all lines of ART, HIV test cost = €6* \| \| \| \| \| \| \| \| \| \| \| \| \| \| Current practice \| 480 \| \| 194.30 \| 0.60 \| \| 70.02 \| \| 90 \| \| \| -- \| \| \| One-time screening \| 490 \| \| 194.31 \| 0.60 \| \| 70.02 \| \| 90 \| \| \| 29,000 \| \| \| *30% increase in cost of all lines of ART, HIV test cost = €6* \| \| \| \| \| \| \| \| \| \| \| \| \| \| Current practice \| 780 \| \| 194.30 \| 0.60 \| \| 70.02 \| \| 140 \| \| \| -- \| \| \| One-time screening \| 790 \| \| 194.31 \| 0.60 \| \| 70.02 \| \| 140 \| \| \| 42,000 \| \| \| *30% decrease in cost of all lines of ART, HIV test cost = €37* \| \| \| \| \| \| \| \| \| \| \| \| \| \| Current practice \| 480 \| \| 194.31 \| 0.60 \| \| 70.03 \| \| 90 \| \| \| -- \| \| \| One-time screening \| 510 \| \| 194.31 \| 0.60 \| \| 70.03 \| \| 90 \| \| \| 73,000 \| \| \| *30% increase in cost of all lines of ART, HIV test cost = €37* \| \| \| \| \| \| \| \| \| \| \| \| \| \| Current practice \| 770 \| \| 194.31 \| 0.60 \| \| \| 70.03 \| \| \| 140 \| \| -- \| \| \| **Table S3. (continued)** \| \| \| \| \| \| \| \| \| \| \| \| \| \| **Testing strategy** \| **Costs (€)^1,2^** \| \| **QALMs, total population^1^** \| \| **Transmissions per HIV-infected person** \| \| **QALMs accrued per transmission^2^** \| \| **Costs accrued per transmission^1^** \| \| **ICER (€/QALY)^3^** \| \| \| One-time screening \| 810 \| \| 194.31 \| 0.60 \| \| 70.03 \| \| 150 \| \| \| 86,000 \| \| \| ***Linkage to care = 30%*** \| \| \| \| \| \| \| \| \| \| \| \| \| \| *30% decrease in cost of all lines of ART, HIV test cost = €6* \| \| \| \| \| \| \| \| \| \| \| \| \| \| Current practice \| 480 \| 194.31 \| \| 0.60 \| \| 70.04 \| \| 90 \| \| \| -- \| \| \| One-time screening \| 500 \| 194.31 \| \| 0.60 \| \| 70.05 \| \| 90 \| \| \| 24,000 \| \| \| *30% increase in cost of all lines of ART, HIV test cost = €6* \| \| \| \| \| \| \| \| \| \| \| \| \| \| Current practice \| 780 \| 194.31 \| \| 0.60 \| \| 70.04 \| \| 140 \| \| \| -- \| \| \| One-time screening \| 800 \| 194.31 \| \| 0.60 \| \| 70.05 \| \| 150 \| \| \| 35,000 \| \| \| *30% decrease in cost of all lines of ART, HIV test cost = €37* \| \| \| \| \| \| \| \| \| \| \| \| \| \| Current practice \| 480 \| 194.30 \| \| 0.60 \| \| 70.10 \| \| 90 \| \| \| -- \| \| \| One-time screening \| 520 \| 194.31 \| \| 0.60 \| \| 70.11 \| \| 100 \| \| \| 49,000 \| \| \| *30% increase in cost of all lines of ART, HIV test cost = €37* \| \| \| \| \| \| \| \| \| \| \| \| \| \| Current practice \| 770 \| 194.30 \| \| 0.60 \| \| 70.10 \| \| 140 \| \| \| -- \| \| \| **Table S3. (continued)** \| \| \| \| \| \| \| \| \| \| \| \| \| \| **Testing strategy** \| **Costs (€)^1,2^** \| **QALMs, total population^1^** \| \| **Transmissions per HIV-infected person** \| \| **QALMs accrued per transmission^2^** \| \| **Costs accrued per transmission^1^** \| \| \| **ICER (€/QALY)^3^** \| \| \| One-time screening \| 820 \| 194.31 \| \| 0.60 \| \| 70.11 \| \| 150 \| \| \| 60,000 \| \| \| ***Linkage to care = 60%*** \| \| \| \| \| \| \| \| \| \| \| \| \| \| *30% decrease in cost of all lines of ART, HIV test cost = €6* \| \| \| \| \| \| \| \| \| \| \| \| \| \| Current practice \| 480 \| 194.30 \| \| 0.60 \| \| 70.10 \| \| 90 \| \| \| -- \| \| \| One-time screening \| 510 \| 194.32 \| \| 0.60 \| \| 70.11 \| \| 100 \| \| \| 22,000 \| \| \| *30% increase in cost of all lines of ART, HIV test cost = €6* \| \| \| \| \| \| \| \| \| \| \| \| \| \| Current practice \| 780 \| 194.30 \| \| 0.60 \| \| 70.10 \| \| 140 \| \| \| -- \| \| \| One-time screening \| 820 \| 194.32 \| \| 0.60 \| \| 70.11 \| \| 150 \| \| \| 34,000 \| \| \| *30% decrease in cost of all lines of ART, HIV test cost = €37* \| \| \| \| \| \| \| \| \| \| \| \| \| \| Current practice \| 480 \| 194.30 \| \| 0.60 \| \| 70.10 \| \| 90 \| \| \| -- \| \| \| One-time screening \| 530 \| 194.32 \| \| 0.6 \| \| 70.11 \| \| 100 \| \| \| 33,000 \| \| \| *30% increase in cost of all lines of ART, HIV test cost = €37* \| \| \| \| \| \| \| \| \| \| \| \| \| \| Current practice \| 770 \| 194.30 \| \| 0.60 \| \| 70.10 \| \| 140 \| \| \| -- \| \| \| **Table S3. (continued)** \| \| \| \| \| \| \| \| \| \| \| \| \| \| **Testing strategy** \| **Costs (€)^1,2^** \| **QALMs, total population^1^** \| \| **Transmissions per HIV-infected person** \| \| **QALMs accrued per transmission^2^** \| \| **Costs accrued per transmission^1^** \| \| \| **ICER (€/QALY)^3^** \| \| \| One-time screening \| 840 \| 194.32 \| \| 0.60 \| \| 70.11 \| \| 160 \| \| \| 44,000 \| \| \| ***Linkage to care = 78.4% (Base case linkage to care)*** \| \| \| \| \| \| \| \| \| \| \| \| \| \| *30% decrease in cost of all lines of ART, HIV test cost = €6* \| \| \| \| \| \| \| \| \| \| \| \| \| \| Current practice \| 480 \| 194.31 \| \| 0.60 \| \| 70.00 \| \| 90 \| \| \| -- \| \| \| One-time screening \| 520 \| 194.32 \| \| 0.60 \| \| 70.01 \| \| 100 \| \| \| 22,000 \| \| \| *30% increase in cost of all lines of ART, HIV test cost = €6* \| \| \| \| \| \| \| \| \| \| \| \| \| \| Current practice \| 770 \| 194.31 \| \| 0.60 \| \| 70.00 \| \| 140 \| \| \| -- \| \| \| One-time screening \| 830 \| 194.32 \| \| 0.60 \| \| 70.01 \| \| 160 \| \| \| 34,000 \| \| \| *30% decrease in cost of all lines of ART, HIV test cost = €37* \| \| \| \| \| \| \| \| \| \| \| \| \| \| Current practice \| 480 \| 194.30 \| \| 0.60 \| \| 70.02 \| \| 90 \| \| \| -- \| \| \| One-time screening \| 540 \| 194.32 \| \| 0.61 \| \| 70.03 \| \| 100 \| \| \| 31,000 \| \| \| *30% increase in cost of all lines of ART, HIV test cost = €37* \| \| \| \| \| \| \| \| \| \| \| \| \| \| Current practice \| 780 \| 194.30 \| \| 0.60 \| \| 70.02 \| \| 140 \| \| \| -- \| \| \| **Table S3. (continued)** \| \|  \| \|  \| \|  \| \|  \| \| \|  \| \| \| **Testing Strategy** \| **Costs (€)^1,2^** \| **QALMs, total population^1^** \| \| **Transmissions per HIV-infected person** \| \| **QALMs accrued per transmission^2^** \| \| **Costs accrued per transmission^1^** \| \| \| **ICER (€/QALY)^3^** \| \| \| One-time screening \| 850 \| 194.32 \| \| 0.61 \| \| 70.03 \| \| 160 \| \| \| 43,000 \| \| \| ***Linkage to care = 100%*** \| \| \| \| \| \| \| \| \| \| \| \| \| \| *30% decrease in cost of all lines of ART, HIV test cost = €6* \| \| \| \| \| \| \| \| \| \| \| \| \| \| Current practice \| 480 \| 194.30 \| \| 0.60 \| \| 69.93 \| \| 90 \| \| \| -- \| \| \| One-time screening \| 530 \| 194.33 \| \| 0.61 \| \| 69.95 \| \| 100 \| \| \| 21,000 \| \| \| *30% increase in cost of all lines of ART, HIV test cost = €6* \| \| \| \| \| \| \| \| \| \| \| \| \| \| Current practice \| 770 \| 194.30 \| \| 0.60 \| \| 69.93 \| \| 140 \| \| \| -- \| \| \| One-time screening \| 850 \| 194.33 \| \| 0.61 \| \| 69.95 \| \| 160 \| \| \| 33,000 \| \| \| *30% decrease in cost of all lines of ART, HIV test cost = €37* \| \| \| \| \| \| \| \| \| \| \| \| \| \| Current practice \| 480 \| 194.30 \| \| 0.60 \| \| 70.02 \| \| 90 \| \| \| -- \| \| \| One-time screening \| 550 \| 194.33 \| \| 0.61 \| \| 70.04 \| \| 110 \| \| \| 29,000 \| \| \| *30% increase in cost of all lines of ART, HIV test cost = €37* \| \| \| \| \| \| \| \| \| \| \| \| \| \| Current practice \| 770 \| 194.30 \| \| 0.60 \| \| 70.02 \| \| 140 \| \| \| -- \| \| \| **Table S3. (continued)** \| \| \| \| \| \| \| \| \| \| \| \| \| \| **Testing Strategy** \| **Costs (€)^1,2^** \| **QALMs, total population^1^** \| \| **Transmissions per HIV-infected person** \| \| **QALMs accrued per transmission^2^** \| \| **Costs accrued per transmission^1^** \| \| \| **ICER (€/QALY)^3^** \| \| \| One-time screening \| 870 \| 194.33 \| \| 0.61 \| \| 70.04 \| \| 160 \| \| \| 41,000 \| \| \| \| --- \| --- \| --- \| --- \| --- \| --- \| --- \| --- \| --- \| --- \| --- \| --- \| --- \| --- \| --- \| --- \| --- \| --- \| --- \| --- \| --- \| --- \| --- \| --- \| --- \| --- \| --- \| --- \| --- \| --- \| --- \| --- \| --- \| --- \| --- \| --- \| --- \| --- \| --- \| --- \| --- \| --- \| --- \| --- \| --- \| --- \| --- \| --- \| --- \| --- \| --- \| --- \| --- \| --- \| --- \| --- \| --- \| --- \| --- \| --- \| --- \| --- \| --- \| --- \| --- \| --- \| --- \| --- \| --- \| --- \| --- \| --- \| --- \| --- \| --- \| --- \| --- \| --- \| --- \| --- \| --- \| --- \| --- \| --- \| --- \| --- \| --- \| --- \| --- \| --- \| --- \| --- \| --- \| --- \| --- \| --- \| --- \| --- \| --- \| --- \| --- \| --- \| --- \| --- \| --- \| --- \| --- \| --- \| --- \| --- \| --- \| --- \| --- \| --- \| --- \| --- \| --- \| --- \| --- \| --- \| --- \| --- \| --- \| --- \| --- \| --- \| --- \| --- \| --- \| --- \| --- \| --- \| --- \| --- \| --- \| --- \| --- \| --- \| --- \| --- \| --- \| --- \| --- \| --- \| --- \| --- \| --- \| --- \| --- \| --- \| --- \| --- \| --- \| --- \| --- \| --- \| --- \| --- \| --- \| --- \| --- \| --- \| --- \| --- \| --- \| --- \| --- \| --- \| --- \| --- \| --- \| --- \| --- \| --- \| --- \| --- \| --- \| --- \| --- \| --- \| --- \| --- \| --- \| --- \| --- \| --- \| --- \| --- \| --- \| --- \| --- \| --- \| --- \| --- \| --- \| --- \| --- \| --- \| --- \| --- \| --- \| --- \| --- \| --- \| --- \| --- \| --- \| --- \| --- \| --- \| --- \| --- \| --- \| --- \| --- \| --- \| --- \| --- \| --- \| --- \| --- \| --- \| --- \| --- \| --- \| --- \| --- \| --- \| --- \| --- \| --- \| --- \| --- \| --- \| --- \| --- \| --- \| --- \| --- \| --- \| --- \| --- \| --- \| --- \| --- \| --- \| --- \| --- \| --- \| --- \| --- \| --- \| --- \| --- \| --- \| --- \| --- \| --- \| --- \| --- \| --- \| --- \| --- \| --- \| --- \| --- \| --- \| --- \| --- \| --- \| --- \| --- \| --- \| --- \| --- \| --- \| --- \| --- \| --- \| --- \| --- \| --- \| --- \| --- \| --- \| --- \| --- \| --- \| --- \| --- \| --- \| --- \| --- \| --- \| --- \| --- \| --- \| --- \| --- \| --- \| --- \| --- \| --- \| --- \| --- \| --- \| --- \| --- \| --- \| --- \| --- \| --- \| --- \| --- \| --- \| --- \| --- \| --- \| --- \| --- \| --- \| --- \| --- \| --- \| --- \| --- \| --- \| --- \| --- \| --- \| --- \| --- \| --- \| --- \| --- \| --- \| --- \| --- \| --- \| --- \| --- \| --- \| --- \| --- \| --- \| --- \| --- \| --- \| --- \| --- \| --- \| --- \| --- \| --- \| --- \| --- \| --- \| --- \| --- \| --- \| --- \| --- \| --- \| --- \| --- \| --- \| --- \| --- \| --- \| --- \| --- \| --- \| --- \| --- \| --- \| --- \| --- \| --- \| --- \| --- \| --- \| --- \| --- \| --- \| --- \| --- \| --- \| --- \| --- \| --- \| --- \| --- \| --- \| --- \| --- \| --- \| --- \| --- \| --- \| --- \| --- \| --- \| --- \| --- \| --- \| --- \| --- \| --- \| --- \| --- \| --- \| --- \| --- \| --- \| --- \| --- \| --- \| --- \| --- \| --- \| --- \| --- \| --- \| --- \| --- \| --- \| --- \| --- \| --- \| --- \| --- \| --- \| --- \| --- \| --- \| --- \| --- \| --- \| --- \| --- \| --- \| --- \| --- \| --- \| --- \| --- \| --- \| --- \| --- \| --- \| --- \| --- \| --- \| --- \| --- \| --- \| --- \| --- \| --- \| --- \| --- \| --- \| --- \| --- \| --- \| --- \| --- \| --- \| --- \| --- \| --- \| --- \| --- \| --- \| --- \| --- \| --- \| --- \| --- \| --- \| --- \| --- \| --- \| --- \| --- \| --- \| --- \| --- \| --- \| --- \| --- \| --- \| --- \| --- \| --- \| --- \| --- \| --- \| --- \| --- \| --- \| --- \| --- \| --- \| --- \| --- \| --- \| --- \| --- \| --- \| --- \| --- \| --- \| --- \| --- \| --- \| --- \| --- \| --- \| --- \| --- \| --- \| --- \| --- \| --- \| --- \| --- \| --- \| --- \| --- \| --- \| --- \| --- \| --- \| --- \| --- \| --- \| --- \| --- \| --- \| --- \| --- \| --- \| --- \| --- \| --- \| --- \| --- \| --- \| --- \| --- \| --- \| --- \| --- \| --- \| --- \| --- \| --- \| --- \| --- \| --- \| --- \| --- \| --- \| --- \| --- \| --- \| --- \| --- \| --- \| --- \| --- \| --- \| --- \| --- \| --- \| --- \| --- \| --- \| --- \| --- \| --- \| --- \| --- \| --- \| --- \| --- \| --- \| --- \| --- \| --- \| --- \| --- \| --- \| --- \| --- \| --- \| --- \| --- \| --- \| --- \| --- \| --- \| --- \| --- \| --- \| --- \| --- \| --- \| --- \| --- \| --- \| --- \| --- \| --- \| --- \| --- \| --- \| --- \| --- \| --- \| --- \| --- \| --- \| --- \| --- \| --- \| --- \| --- \| --- \| --- \| --- \| --- \| --- \| --- \| --- \| --- \| --- \| --- \| --- \| --- \| --- \| --- \| --- \| --- \| --- \| --- \| --- \| --- \| --- \| --- \| --- \| --- \| --- \| --- \| --- \| --- \| --- \| --- \| --- \| --- \| --- \| --- \| --- \| --- \| --- \| --- \| --- \| --- \| --- \| --- \| --- \| --- \| --- \| --- \| --- \| --- \| --- \| --- \| --- \| --- \| --- \| --- \| --- \| --- \| --- \| --- \| --- \| --- \| --- \| --- \| --- \| --- \| --- \| --- \| --- \| --- \| --- \| --- \| --- \| --- \| --- \| --- \| --- \| --- \| --- \| --- \| --- \| --- \| --- \| --- \| --- \| --- \| --- \| --- \| --- \| --- \| --- \| --- \| --- \| --- \| --- \| --- \| --- \| --- \| --- \| --- \| --- \| --- \| --- \| --- \| --- \| --- \| --- \| --- \| --- \| --- \| --- \| --- \| --- \| --- \| --- \| --- \| --- \| --- \| --- \| --- \| --- \| --- \| --- \| --- \| --- \| --- \| --- \| --- \| --- \| --- \| --- \| --- \| --- \| --- \| --- \| --- \| --- \| --- \| --- \| --- \| --- \| --- \| --- \| --- \| --- \| --- \| --- \| --- \| --- \| --- \| --- \| --- \| --- \| --- \| --- \| --- \| --- \| --- \| --- \| --- \| --- \| --- \| --- \| --- \| --- \| --- \| --- \| --- \| --- \| --- \| --- \| --- \| --- \| --- \| --- \| --- \| --- \| --- \| --- \| --- \| --- \| --- \| --- \| --- \| --- \| --- \| --- \| --- \| --- \| --- \| --- \| --- \| --- \| --- \| --- \| --- \| --- \| --- \| --- \| --- \| --- \| --- \| --- \| --- \| --- \| --- \| --- \| --- \| --- \| --- \| --- \| --- \| --- \| --- \| --- \| --- \| --- \| --- \| --- \| --- \| --- \| --- \| --- \| --- \| --- \| --- \| --- \| --- \| --- \| --- \| --- \| --- \| --- \| --- \| --- \| --- \| --- \| --- \| --- \| --- \| --- \| --- \| --- \| --- \| --- \| --- \| --- \| --- \| --- \| --- \| --- \| --- \| --- \| --- \| --- \| --- \| --- \| --- \| --- \| --- \| --- \| --- \| --- \| --- \| --- \| --- \| --- \| --- \| --- \| --- \| --- \| --- \| --- \| --- \| --- \| --- \| --- \| --- \| --- \| --- \| --- \| --- \| --- \| --- \| --- \| --- \| --- \| --- \| --- \| --- \| --- \| --- \| --- \| --- \| --- \| --- \| --- \| --- \| --- \| --- \| --- \| --- \| --- \| --- \| --- \| --- \| --- \| --- \| --- \| --- \| --- \| --- \| --- \| --- \| --- \| --- \| --- \| --- \| --- \| --- \| --- \| --- \| --- \| --- \| --- \| --- \| --- \| --- \| --- \| --- \| --- \| --- \| --- \| --- \| --- \| --- \| --- \| --- \| --- \| --- \| --- \| --- \| --- \| --- \| --- \| --- \| --- \| --- \| --- \| --- \| --- \| --- \| --- \| --- \| --- \| --- \| --- \| --- \| --- \| --- \| --- \| --- \| --- \| \| \| --- \| --- \| --- \| --- \| --- \| --- \| --- \| --- \| --- \| --- \| --- \| --- \| --- \| --- \| --- \| --- \| --- \| --- \| --- \| --- \| --- \| --- \| --- \| --- \| --- \| --- \| --- \| --- \| --- \| --- \| --- \| --- \| --- \| --- \| --- \| --- \| --- \| --- \| --- \| --- \| --- \| --- \| --- \| --- \| --- \| --- \| --- \| --- \| --- \| --- \| --- \| --- \| --- \| --- \| --- \| --- \| --- \| --- \| --- \| --- \| --- \| --- \| --- \| --- \| --- \| --- \| --- \| --- \| --- \| --- \| --- \| --- \| --- \| --- \| --- \| --- \| --- \| --- \| --- \| --- \| --- \| --- \| --- \| --- \| --- \| --- \| --- \| --- \| --- \| --- \| --- \| --- \| --- \| --- \| --- \| --- \| --- \| --- \| --- \| --- \| --- \| --- \| --- \| --- \| --- \| --- \| --- \| --- \| --- \| --- \| --- \| --- \| --- \| --- \| --- \| --- \| --- \| --- \| --- \| --- \| --- \| --- \| --- \| --- \| --- \| --- \| --- \| --- \| --- \| --- \| --- \| --- \| --- \| --- \| --- \| --- \| --- \| --- \| --- \| --- \| --- \| --- \| --- \| --- \| --- \| --- \| --- \| --- \| --- \| --- \| --- \| --- \| --- \| --- \| --- \| --- \| --- \| --- \| --- \| --- \| --- \| --- \| --- \| --- \| --- \| --- \| --- \| --- \| --- \| --- \| --- \| --- \| --- \| --- \| --- \| --- \| --- \| --- \| --- \| --- \| --- \| --- \| --- \| --- \| --- \| --- \| --- \| --- \| --- \| --- \| --- \| --- \| --- \| --- \| --- \| --- \| --- \| --- \| --- \| --- \| --- \| --- \| --- \| --- \| --- \| --- \| --- \| --- \| --- \| --- \| --- \| --- \| --- \| --- \| --- \| --- \| --- \| --- \| --- \| --- \| --- \| --- \| --- \| --- \| --- \| --- \| --- \| --- \| --- \| --- \| --- \| --- \| --- \| --- \| --- \| --- \| --- \| --- \| --- \| --- \| --- \| --- \| --- \| --- \| --- \| --- \| --- \| --- \| --- \| --- \| --- \| --- \| --- \| --- \| --- \| --- \| --- \| --- \| --- \| --- \| --- \| --- \| --- \| --- \| --- \| --- \| --- \| --- \| --- \| --- \| --- \| --- \| --- \| --- \| --- \| --- \| --- \| --- \| --- \| --- \| --- \| --- \| --- \| --- \| --- \| --- \| --- \| --- \| --- \| --- \| --- \| --- \| --- \| --- \| --- \| --- \| --- \| --- \| --- \| --- \| --- \| --- \| --- \| --- \| --- \| --- \| --- \| --- \| --- \| --- \| --- \| --- \| --- \| --- \| --- \| --- \| --- \| --- \| --- \| --- \| --- \| --- \| --- \| --- \| --- \| --- \| --- \| --- \| --- \| --- \| --- \| --- \| --- \| --- \| --- \| --- \| --- \| --- \| --- \| --- \| --- \| --- \| --- \| --- \| --- \| --- \| --- \| --- \| --- \| --- \| --- \| --- \| --- \| --- \| --- \| --- \| --- \| --- \| --- \| --- \| --- \| --- \| --- \| --- \| --- \| --- \| --- \| --- \| --- \| --- \| --- \| --- \| --- \| --- \| --- \| --- \| --- \| --- \| --- \| --- \| --- \| --- \| --- \| --- \| --- \| --- \| --- \| --- \| --- \| --- \| --- \| --- \| --- \| --- \| --- \| --- \| --- \| --- \| --- \| --- \| --- \| --- \| --- \| --- \| --- \| --- \| --- \| --- \| --- \| --- \| --- \| --- \| --- \| --- \| --- \| --- \| --- \| --- \| --- \| --- \| --- \| --- \| --- \| --- \| --- \| --- \| --- \| --- \| --- \| --- \| --- \| --- \| --- \| --- \| --- \| --- \| --- \| --- \| --- \| --- \| --- \| --- \| --- \| --- \| --- \| --- \| --- \| --- \| --- \| --- \| --- \| --- \| --- \| --- \| --- \| --- \| --- \| --- \| --- \| --- \| --- \| --- \| --- \| --- \| --- \| --- \| --- \| --- \| --- \| --- \| --- \| --- \| --- \| --- \| --- \| --- \| --- \| --- \| --- \| --- \| --- \| --- \| --- \| --- \| --- \| --- \| --- \| --- \| --- \| --- \| --- \| --- \| --- \| --- \| --- \| --- \| --- \| --- \| --- \| --- \| --- \| --- \| --- \| --- \| --- \| --- \| --- \| --- \| --- \| --- \| --- \| --- \| --- \| --- \| --- \| --- \| --- \| --- \| --- \| --- \| --- \| --- \| --- \| --- \| --- \| --- \| --- \| --- \| --- \| --- \| --- \| --- \| --- \| --- \| --- \| --- \| --- \| --- \| --- \| --- \| --- \| --- \| --- \| --- \| --- \| --- \| --- \| --- \| --- \| --- \| --- \| --- \| --- \| --- \| --- \| --- \| --- \| --- \| --- \| --- \| --- \| --- \| --- \| --- \| --- \| --- \| --- \| --- \| --- \| --- \| --- \| --- \| --- \| --- \| --- \| --- \| --- \| --- \| --- \| --- \| --- \| --- \| --- \| --- \| --- \| --- \| --- \| --- \| --- \| --- \| --- \| --- \| --- \| --- \| --- \| --- \| --- \| --- \| --- \| --- \| --- \| --- \| --- \| --- \| --- \| --- \| --- \| --- \| --- \| --- \| --- \| --- \| --- \| --- \| --- \| --- \| --- \| --- \| --- \| --- \| --- \| --- \| --- \| --- \| --- \| --- \| --- \| --- \| --- \| --- \| --- \| --- \| --- \| --- \| --- \| --- \| --- \| --- \| --- \| --- \| --- \| --- \| --- \| --- \| --- \| --- \| --- \| --- \| --- \| --- \| --- \| --- \| --- \| --- \| --- \| --- \| --- \| --- \| --- \| --- \| --- \| --- \| --- \| --- \| --- \| --- \| --- \| --- \| --- \| --- \| --- \| --- \| --- \| --- \| --- \| --- \| --- \| --- \| --- \| --- \| --- \| --- \| --- \| --- \| --- \| --- \| --- \| --- \| --- \| --- \| --- \| --- \| --- \| --- \| --- \| --- \| --- \| --- \| --- \| --- \| --- \| --- \| --- \| --- \| --- \| --- \| --- \| --- \| --- \| --- \| --- \| --- \| --- \| --- \| --- \| --- \| --- \| --- \| --- \| --- \| --- \| --- \| --- \| --- \| --- \| --- \| --- \| --- \| --- \| --- \| --- \| --- \| --- \| --- \| --- \| --- \| --- \| --- \| --- \| --- \| --- \| --- \| --- \| --- \| --- \| --- \| --- \| --- \| --- \| --- \| --- \| --- \| --- \| --- \| --- \| --- \| --- \| --- \| --- \| --- \| --- \| --- \| --- \| --- \| --- \| --- \| --- \| --- \| --- \| --- \| --- \| --- \| --- \| --- \| --- \| --- \| --- \| --- \| --- \| --- \| --- \| --- \| --- \| --- \| --- \| --- \| --- \| --- \| --- \| --- \| --- \| --- \| --- \| --- \| --- \| --- \| --- \| --- \| --- \| --- \| --- \| --- \| --- \| --- \| --- \| --- \| --- \| --- \| --- \| --- \| --- \| --- \| --- \| --- \| --- \| --- \| --- \| --- \| --- \| --- \| --- \| --- \| --- \| --- \| --- \| --- \| --- \| --- \| --- \| --- \| --- \| --- \| --- \| --- \| --- \| --- \| --- \| --- \| --- \| --- \| --- \| --- \| --- \| --- \| --- \| --- \| --- \| --- \| --- \| --- \| --- \| --- \| --- \| --- \| --- \| --- \| --- \| --- \| --- \| --- \| --- \| --- \| --- \| --- \| --- \| --- \| --- \| --- \| --- \| --- \| --- \| --- \| --- \| --- \| --- \| --- \| --- \| --- \| --- \| --- \| --- \| --- \| --- \| --- \| --- \| --- \| --- \| --- \| --- \| --- \| --- \| --- \| --- \| --- \| --- \| --- \| --- \| --- \| --- \| --- \| --- \| --- \| --- \| --- \| --- \| --- \| --- \| --- \| --- \| --- \| --- \| --- \| --- \| --- \| --- \| --- \| --- \| --- \| --- \| --- \| --- \| --- \| --- \| --- \| --- \| --- \| --- \| --- \| --- \| --- \| --- \| --- \| --- \| --- \| --- \| --- \| --- \| --- \| --- \| --- \| --- \| --- \| --- \| --- \| --- \| --- \| --- \| --- \| --- \| --- \| --- \| --- \| --- \| --- \| --- \| --- \| --- \| --- \| --- \| --- \| --- \| --- \| --- \| --- \| --- \| --- \| --- \| --- \| --- \| --- \| --- \| --- \| --- \| --- \| --- \| --- \| --- \| --- \| --- \| --- \| --- \| --- \| --- \| --- \| --- \| --- \| --- \| --- \| --- \| --- \| --- \| \| \| --- \| --- \| --- \| --- \| --- \| --- \| --- \| --- \| --- \| --- \| --- \| --- \| --- \| --- \| --- \| --- \| --- \| --- \| --- \| --- \| --- \| --- \| --- \| --- \| --- \| --- \| --- \| --- \| --- \| --- \| --- \| --- \| --- \| --- \| --- \| --- \| --- \| --- \| --- \| --- \| --- \| --- \| --- \| --- \| --- \| --- \| --- \| --- \| --- \| --- \| --- \| --- \| --- \| --- \| --- \| --- \| --- \| --- \| --- \| --- \| --- \| --- \| --- \| --- \| --- \| --- \| --- \| --- \| --- \| --- \| --- \| --- \| --- \| --- \| --- \| --- \| --- \| --- \| --- \| --- \| --- \| --- \| --- \| --- \| --- \| --- \| --- \| --- \| --- \| --- \| --- \| --- \| --- \| --- \| --- \| --- \| --- \| --- \| --- \| --- \| --- \| --- \| --- \| --- \| --- \| --- \| --- \| --- \| --- \| --- \| --- \| --- \| --- \| --- \| --- \| --- \| --- \| --- \| --- \| --- \| --- \| --- \| --- \| --- \| --- \| --- \| --- \| --- \| --- \| --- \| --- \| --- \| --- \| --- \| --- \| --- \| --- \| --- \| --- \| --- \| --- \| --- \| --- \| --- \| --- \| --- \| --- \| --- \| --- \| --- \| --- \| --- \| --- \| --- \| --- \| --- \| --- \| --- \| --- \| --- \| --- \| --- \| --- \| --- \| --- \| --- \| --- \| --- \| --- \| --- \| --- \| --- \| --- \| --- \| --- \| --- \| --- \| --- \| --- \| --- \| --- \| --- \| --- \| --- \| --- \| --- \| --- \| --- \| --- \| --- \| --- \| --- \| --- \| --- \| --- \| --- \| --- \| --- \| --- \| --- \| --- \| --- \| --- \| --- \| --- \| --- \| --- \| --- \| --- \| --- \| --- \| --- \| --- \| --- \| --- \| --- \| --- \| --- \| --- \| --- \| --- \| --- \| --- \| --- \| --- \| --- \| --- \| --- \| --- \| --- \| --- \| --- \| --- \| --- \| --- \| --- \| --- \| --- \| --- \| --- \| --- \| --- \| --- \| --- \| --- \| --- \| --- \| --- \| --- \| --- \| --- \| --- \| --- \| --- \| --- \| --- \| --- \| --- \| --- \| --- \| --- \| --- \| --- \| --- \| --- \| --- \| --- \| --- \| --- \| --- \| --- \| --- \| --- \| --- \| --- \| --- \| --- \| --- \| --- \| --- \| --- \| --- \| --- \| --- \| --- \| --- \| --- \| --- \| --- \| --- \| --- \| --- \| --- \| --- \| --- \| --- \| --- \| --- \| --- \| --- \| --- \| --- \| --- \| --- \| --- \| --- \| --- \| --- \| --- \| --- \| --- \| --- \| --- \| --- \| --- \| --- \| --- \| --- \| --- \| --- \| --- \| --- \| --- \| --- \| --- \| --- \| --- \| --- \| --- \| --- \| --- \| --- \| --- \| --- \| --- \| --- \| --- \| --- \| --- \| --- \| --- \| --- \| --- \| --- \| --- \| --- \| --- \| --- \| --- \| --- \| --- \| --- \| --- \| --- \| --- \| --- \| --- \| --- \| --- \| --- \| --- \| --- \| --- \| --- \| --- \| --- \| --- \| --- \| --- \| --- \| --- \| --- \| --- \| --- \| --- \| --- \| --- \| --- \| --- \| --- \| --- \| --- \| --- \| --- \| --- \| --- \| --- \| --- \| --- \| --- \| --- \| --- \| --- \| --- \| --- \| --- \| --- \| --- \| --- \| --- \| --- \| --- \| --- \| --- \| --- \| --- \| --- \| --- \| --- \| --- \| --- \| --- \| --- \| --- \| --- \| --- \| --- \| --- \| --- \| --- \| --- \| --- \| --- \| --- \| --- \| --- \| --- \| --- \| --- \| --- \| --- \| --- \| --- \| --- \| --- \| --- \| --- \| --- \| --- \| --- \| --- \| --- \| --- \| --- \| --- \| --- \| --- \| --- \| --- \| --- \| --- \| --- \| --- \| --- \| --- \| --- \| --- \| --- \| --- \| --- \| --- \| --- \| --- \| --- \| --- \| --- \| --- \| --- \| --- \| --- \| --- \| --- \| --- \| --- \| --- \| --- \| --- \| --- \| --- \| --- \| --- \| --- \| --- \| --- \| --- \| --- \| --- \| --- \| --- \| --- \| --- \| --- \| --- \| --- \| --- \| --- \| --- \| --- \| --- \| --- \| --- \| --- \| --- \| --- \| --- \| --- \| --- \| --- \| --- \| --- \| --- \| --- \| --- \| --- \| --- \| --- \| --- \| --- \| --- \| --- \| --- \| --- \| --- \| --- \| --- \| --- \| --- \| --- \| --- \| --- \| --- \| --- \| --- \| --- \| --- \| --- \| --- \| --- \| --- \| --- \| --- \| --- \| --- \| --- \| --- \| --- \| --- \| --- \| --- \| --- \| --- \| --- \| --- \| --- \| --- \| --- \| --- \| --- \| --- \| --- \| --- \| --- \| --- \| --- \| --- \| --- \| --- \| --- \| --- \| --- \| --- \| --- \| --- \| --- \| --- \| --- \| --- \| --- \| --- \| --- \| --- \| --- \| --- \| --- \| --- \| --- \| --- \| --- \| --- \| --- \| --- \| --- \| --- \| --- \| --- \| --- \| --- \| --- \| --- \| --- \| --- \| --- \| --- \| --- \| --- \| --- \| --- \| --- \| --- \| --- \| --- \| --- \| --- \| --- \| --- \| --- \| --- \| --- \| --- \| --- \| --- \| --- \| --- \| --- \| --- \| --- \| --- \| --- \| --- \| --- \| --- \| --- \| --- \| --- \| --- \| --- \| --- \| --- \| --- \| --- \| --- \| --- \| --- \| --- \| --- \| --- \| --- \| --- \| --- \| --- \| --- \| --- \| --- \| --- \| --- \| --- \| --- \| --- \| --- \| --- \| --- \| --- \| --- \| --- \| --- \| --- \| --- \| --- \| --- \| --- \| --- \| --- \| --- \| --- \| --- \| --- \| --- \| --- \| --- \| --- \| --- \| --- \| --- \| --- \| --- \| --- \| --- \| --- \| --- \| --- \| --- \| --- \| --- \| --- \| --- \| --- \| --- \| --- \| --- \| --- \| --- \| --- \| --- \| --- \| --- \| --- \| --- \| --- \| --- \| --- \| --- \| --- \| --- \| --- \| --- \| --- \| --- \| --- \| --- \| --- \| --- \| --- \| --- \| --- \| --- \| --- \| --- \| --- \| --- \| --- \| --- \| --- \| --- \| --- \| --- \| --- \| --- \| --- \| --- \| --- \| --- \| --- \| --- \| --- \| --- \| --- \| --- \| --- \| --- \| --- \| --- \| --- \| --- \| --- \| --- \| --- \| --- \| --- \| --- \| --- \| --- \| --- \| --- \| --- \| --- \| --- \| --- \| --- \| --- \| --- \| --- \| --- \| --- \| --- \| --- \| --- \| --- \| --- \| --- \| --- \| --- \| --- \| --- \| --- \| --- \| --- \| --- \| --- \| --- \| --- \| --- \| --- \| --- \| --- \| --- \| --- \| --- \| --- \| --- \| --- \| --- \| --- \| --- \| --- \| --- \| --- \| --- \| --- \| --- \| --- \| --- \| --- \| --- \| --- \| --- \| --- \| --- \| --- \| --- \| --- \| --- \| --- \| --- \| --- \| --- \| --- \| --- \| --- \| --- \| --- \| --- \| --- \| --- \| --- \| --- \| --- \| --- \| --- \| --- \| --- \| --- \| --- \| --- \| --- \| --- \| --- \| --- \| --- \| --- \| --- \| --- \| --- \| --- \| --- \| --- \| --- \| --- \| --- \| --- \| --- \| --- \| --- \| --- \| --- \| --- \| --- \| --- \| --- \| --- \| --- \| --- \| --- \| --- \| --- \| --- \| --- \| --- \| --- \| --- \| --- \| --- \| --- \| --- \| --- \| --- \| --- \| --- \| --- \| --- \| --- \| --- \| --- \| --- \| --- \| --- \| --- \| --- \| --- \| --- \| --- \| --- \| --- \| --- \| --- \| --- \| --- \| --- \| --- \| --- \| --- \| --- \| --- \| --- \| --- \| --- \| --- \| --- \| --- \| --- \| --- \| --- \| --- \| --- \| --- \| --- \| --- \| --- \| --- \| --- \| --- \| --- \| --- \| --- \| --- \| --- \| --- \| --- \| --- \| --- \| --- \| --- \| --- \| --- \| --- \| --- \| --- \| --- \| --- \| --- \| --- \| --- \| --- \| --- \| --- \| --- \| --- \| --- \| --- \| --- \| --- \| --- \| --- \| --- \| --- \| --- \| --- \| --- \| --- \| --- \| --- \| --- \| --- \| --- \| --- \| --- \| --- \| --- \| --- \| --- \| --- \| --- \| --- \| --- \| --- \| --- \| --- \| --- \| --- \| --- \| --- \| --- \| --- \| |
| --- | --- | --- | --- | --- | --- | --- | --- | --- | --- | --- | --- | --- | --- | --- | --- | --- | --- | --- | --- | --- | --- | --- | --- | --- | --- | --- | --- | --- | --- | --- | --- | --- | --- | --- | --- | --- | --- | --- | --- | --- | --- | --- | --- | --- | --- | --- | --- | --- | --- | --- | --- | --- | --- | --- | --- | --- | --- | --- | --- | --- | --- | --- | --- | --- | --- | --- | --- | --- | --- | --- | --- | --- | --- | --- | --- | --- | --- | --- | --- | --- | --- | --- | --- | --- | --- | --- | --- | --- | --- | --- | --- | --- | --- | --- | --- | --- | --- | --- | --- | --- | --- | --- | --- | --- | --- | --- | --- | --- | --- | --- | --- | --- | --- | --- | --- | --- | --- | --- | --- | --- | --- | --- | --- | --- | --- | --- | --- | --- | --- | --- | --- | --- | --- | --- | --- | --- | --- | --- | --- | --- | --- | --- | --- | --- | --- | --- | --- | --- | --- | --- | --- | --- | --- | --- | --- | --- | --- | --- | --- | --- | --- | --- | --- | --- | --- | --- | --- | --- | --- | --- | --- | --- | --- | --- | --- | --- | --- | --- | --- | --- | --- | --- | --- | --- | --- | --- | --- | --- | --- | --- | --- | --- | --- | --- | --- | --- | --- | --- | --- | --- | --- | --- | --- | --- | --- | --- | --- | --- | --- | --- | --- | --- | --- | --- | --- | --- | --- | --- | --- | --- | --- | --- | --- | --- | --- | --- | --- | --- | --- | --- | --- | --- | --- | --- | --- | --- | --- | --- | --- | --- | --- | --- | --- | --- | --- | --- | --- | --- | --- | --- | --- | --- | --- | --- | --- | --- | --- | --- | --- | --- | --- | --- | --- | --- | --- | --- | --- | --- | --- | --- | --- | --- | --- | --- | --- | --- | --- | --- | --- | --- | --- | --- | --- | --- | --- | --- | --- | --- | --- | --- | --- | --- | --- | --- | --- | --- | --- | --- | --- | --- | --- | --- | --- | --- | --- | --- | --- | --- | --- | --- | --- | --- | --- | --- | --- | --- | --- | --- | --- | --- | --- | --- | --- | --- | --- | --- | --- | --- | --- | --- | --- | --- | --- | --- | --- | --- | --- | --- | --- | --- | --- | --- | --- | --- | --- | --- | --- | --- | --- | --- | --- | --- | --- | --- | --- | --- | --- | --- | --- | --- | --- | --- | --- | --- | --- | --- | --- | --- | --- | --- | --- | --- | --- | --- | --- | --- | --- | --- | --- | --- | --- | --- | --- | --- | --- | --- | --- | --- | --- | --- | --- | --- | --- | --- | --- | --- | --- | --- | --- | --- | --- | --- | --- | --- | --- | --- | --- | --- | --- | --- | --- | --- | --- | --- | --- | --- | --- | --- | --- | --- | --- | --- | --- | --- | --- | --- | --- | --- | --- | --- | --- | --- | --- | --- | --- | --- | --- | --- | --- | --- | --- | --- | --- | --- | --- | --- | --- | --- | --- | --- | --- | --- | --- | --- | --- | --- | --- | --- | --- | --- | --- | --- | --- | --- | --- | --- | --- | --- | --- | --- | --- | --- | --- | --- | --- | --- | --- | --- | --- | --- | --- | --- | --- | --- | --- | --- | --- | --- | --- | --- | --- | --- | --- | --- | --- | --- | --- | --- | --- | --- | --- | --- | --- | --- | --- | --- | --- | --- | --- | --- | --- | --- | --- | --- | --- | --- | --- | --- | --- | --- | --- | --- | --- | --- | --- | --- | --- | --- | --- | --- | --- | --- | --- | --- | --- | --- | --- | --- | --- | --- | --- | --- | --- | --- | --- | --- | --- | --- | --- | --- | --- | --- | --- | --- | --- | --- | --- | --- | --- | --- | --- | --- | --- | --- | --- | --- | --- | --- | --- | --- | --- | --- | --- | --- | --- | --- | --- | --- | --- | --- | --- | --- | --- | --- | --- | --- | --- | --- | --- | --- | --- | --- | --- | --- | --- | --- | --- | --- | --- | --- | --- | --- | --- | --- | --- | --- | --- | --- | --- | --- | --- | --- | --- | --- | --- | --- | --- | --- | --- | --- | --- | --- | --- | --- | --- | --- | --- | --- | --- | --- | --- | --- | --- | --- | --- | --- | --- | --- | --- | --- | --- | --- | --- | --- | --- | --- | --- | --- | --- | --- | --- | --- | --- | --- | --- | --- | --- | --- | --- | --- | --- | --- | --- | --- | --- | --- | --- | --- | --- | --- | --- | --- | --- | --- | --- | --- | --- | --- | --- | --- | --- | --- | --- | --- | --- | --- | --- | --- | --- | --- | --- | --- | --- | --- | --- | --- | --- | --- | --- | --- | --- | --- | --- | --- | --- | --- | --- | --- | --- | --- | --- | --- | --- | --- | --- | --- | --- | --- | --- | --- | --- | --- | --- | --- | --- | --- | --- | --- | --- | --- | --- | --- | --- | --- | --- | --- | --- | --- | --- | --- | --- | --- | --- | --- | --- | --- | --- | --- | --- | --- | --- | --- | --- | --- | --- | --- | --- | --- | --- | --- | --- | --- | --- | --- | --- | --- | --- | --- | --- | --- | --- | --- | --- | --- | --- | --- | --- | --- | --- | --- | --- | --- | --- | --- | --- | --- | --- | --- | --- | --- | --- | --- | --- | --- | --- | --- | --- | --- | --- | --- | --- | --- | --- | --- | --- | --- | --- | --- | --- | --- | --- | --- | --- | --- | --- | --- | --- | --- | --- | --- | --- | --- | --- | --- | --- | --- | --- | --- | --- | --- | --- | --- | --- | --- | --- | --- | --- | --- | --- | --- | --- | --- | --- | --- | --- | --- | --- | --- | --- | --- | --- | --- | --- | --- | --- | --- | --- | --- | --- | --- | --- | --- | --- | --- | --- | --- | --- | --- | --- | --- | --- | --- | --- | --- | --- | --- | --- | --- | --- | --- | --- | --- | --- | --- | --- | --- | --- | --- | --- | --- | --- | --- | --- | --- | --- | --- | --- | --- | --- | --- | --- | --- | --- | --- | --- | --- | --- | --- | --- | --- | --- | --- | --- | --- | --- | --- | --- | --- | --- | --- | --- | --- | --- | --- | --- | --- | --- | --- | --- | --- | --- | --- | --- | --- | --- | --- | --- | --- | --- | --- | --- | --- | --- | --- | --- | --- | --- | --- | --- | --- | --- | --- | --- | --- | --- | --- | --- | --- | --- | --- | --- | --- | --- | --- | --- | --- | --- | --- | --- | --- | --- | --- | --- | --- | --- | --- | --- | --- | --- | --- | --- | --- | --- | --- | --- | --- | --- | --- | --- | --- | --- | --- |

1. Costs are rounded to the nearest €10 and discounted at 5% per annum.
2. QALMs are discounted at 5% per annum.
3. Incremental cost-effectiveness with secondary transmission = [(cost per person in strategy 2 + cost accrued per transmission in strategy 2) – (cost per person in strategy 1 + cost accrued per transmission in strategy 1)]/[(QALMs per person in strategy 2 + QALMs accrued per transmission in strategy 2) – (QALMs per person in strategy 1 + QALMs accrued per transmission in strategy 1)]. ICERs are rounded to the nearest €1,000/QALY.

**Table S4. Multi-way sensitivity analysis impact of HIV prevalence, HIV testing costs and ART costs on one-time, national, routine screening**

| **Testing strategy** | **Costs (€)^1,2^** | | **QALMs, total population^1^** | | **Transmissions per HIV-infected person** | | **QALMs accrued per transmission^2^** | | **Costs accrued per transmission^1^** | **ICER (€/QALY)^3^** |
| --- | --- | --- | --- | --- | --- | --- | --- | --- | --- | --- |
| ***Overall HIV prevalence = 0.10%*** | | | | | | | | | | |
| *30% decrease in cost of all lines of ART, HIV test cost = €6* | | | | | | | | | | |
| Current practice | 350 | | 194.43 | 0.60 | | 51.37 | | 60 | | -- |
| One-time screening | 360 | | 194.43 | 0.60 | | 51.38 | | 70 | | 27,000 |
| *30% increase in cost of all lines of ART, HIV test cost = €6* | | | | | | | | | | |
| Current practice | 560 | | 194.43 | 0.60 | | 51.37 | | 100 | | -- |
| One-time screening | 580 | | 194.43 | 0.60 | | 51.38 | | 120 | | 39,000 |
| *30% decrease in cost of all lines of ART, HIV test cost = €37* | | | | | | | | | | |
| Current practice | 350 | | 194.43 | 0.60 | | 51.33 | | 60 | | -- |
| One-time screening | 380 | | 194.43 | 0.60 | | 51.34 | | 70 | | 51,000 |
| *30% increase in cost of all lines of ART, HIV test cost = €37* | | | | | | | | | | |
| Current practice | 560 | | 194.43 | 0.60 | | 51.33 | | 100 | | -- |
| **Table S4. (continued)** | | | | | | | | | | |
| **Testing strategy** | **Costs (€)^1,2^** | | **QALMs, total population^1^** | | **Transmissions per HIV-infected person** | | **QALMs accrued per transmission^2^** | | **Costs accrued per transmission^1^** | **ICER (€/QALY)^3^** |
| One-time screening | 600 | | 194.43 | 0.60 | | 51.34 | | 120 | | 61,000 |
| ***Overall HIV prevalence = 0.20%*** | | | | | | | | | | |
| *30% decrease in cost of all lines of ART, HIV test cost = €6* | | | | | | | | | | |
| Current practice | 380 | | 194.40 | 0.60 | | 55.64 | | 70 | | -- |
| One-time screening | 400 | | 194.40 | 0.60 | | 55.65 | | 80 | | 23,000 |
| *30% increase in cost of all lines of ART, HIV test cost = €6* | | | | | | | | | | |
| Current practice | 610 | | 194.40 | 0.60 | | 55.64 | | 110 | | -- |
| One-time screening | 640 | | 194.40 | 0.60 | | 55.65 | | 130 | | 35,000 |
| *30% decrease in cost of all lines of ART, HIV test cost = €37* | | | | | | | | | | |
| Current practice | 380 | | 194.40 | 0.60 | | 55.64 | | 70 | | -- |
| One-time screening | 420 | | 194.40 | 0.60 | | 55.65 | | 80 | | 44,000 |
| *30% increase in cost of all lines of ART, HIV test cost = €37* | | | | | | | | | | |
| Current practice | 610 | | 194.40 | 0.60 | | 55.64 | | 110 | | -- |
| **Table S4. (continued)** | | | | | | | | | | |
| **Testing strategy** | **Costs (€)^1,2^** | | **QALMs, total population^1^** | | **Transmissions per HIV-infected person** | | **QALMs accrued per transmission^2^** | | **Costs accrued per transmission^1^** | **ICER (€/QALY)^3^** |
| One-time screening | 660 | | 194.40 | 0.60 | | 55.65 | | 130 | | 56,000 |
| ***Overall HIV prevalence = 0.30%*** | | | | | | | | | | |
| *30% decrease in cost of all lines of ART, HIV test cost = €6* | | | | | | | | | | |
| Current practice | 410 | 194.37 | | 0.60 | | 59.89 | | 70 | | -- |
| One-time screening | 430 | 194.38 | | 0.60 | | 59.90 | | 90 | | 23,000 |
| *30% increase in cost of all lines of ART, HIV test cost = €6* | | | | | | | | | | |
| Current practice | 660 | 194.37 | | 0.60 | | 59.89 | | 120 | | -- |
| One-time screening | 700 | 194.38 | | 0.60 | | 59.90 | | 140 | | 35,000 |
| *30% decrease in cost of all lines of ART, HIV test cost = €37* | | | | | | | | | | |
| Current practice | 410 | 194.37 | | 0.60 | | 59.95 | | 70 | | -- |
| One-time screening | 450 | 194.38 | | 0.60 | | 59.95 | | 90 | | 39,000 |
| *30% increase in cost of all lines of ART, HIV test cost = €37* | | | | | | | | | | |
| Current practice | 660 | 194.37 | | 0.60 | | 59.95 | | 120 | | -- |
| **Table S4. (continued)** | | | | | | | | | | |
| **Testing strategy** | **Costs (€)^1,2^** | | **QALMs, total population^1^** | | **Transmissions per HIV-infected person** | | **QALMs accrued per transmission^2^** | | **Costs accrued per transmission^1^** | **ICER (€/QALY)^3^** |
| One-time screening | 720 | 194.38 | | 0.60 | | 59.95 | | 140 | | 51,000 |
| ***Overall HIV prevalence = 0.53% (Base case HIV prevalence)*** | | | | | | | | | | |
| *30% decrease in cost of all lines of ART, HIV test cost = €6* | | | | | | | | | | |
| Current practice | 480 | 194.31 | | 0.60 | | 70.00 | | 90 | | -- |
| One-time screening | 520 | 194.32 | | 0.60 | | 70.01 | | 100 | | 22,000 |
| *30% increase in cost of all lines of ART, HIV test cost = €6* | | | | | | | | | | |
| Current practice | 770 | 194.31 | | 0.60 | | 70.00 | | 140 | | -- |
| One-time screening | 830 | 194.32 | | 0.60 | | 70.01 | | 160 | | 34,000 |
| *30% decrease in cost of all lines of ART, HIV test cost = €37* | | | | | | | | | | |
| Current practice | 480 | 194.30 | | 0.60 | | 70.02 | | 90 | | -- |
| One-time screening | 540 | 194.32 | | 0.61 | | 70.03 | | 100 | | 31,000 |
| *30% increase in cost of all lines of ART, HIV test cost = €37* | | | | | | | | | | |
| Current practice | 780 | 194.30 | | 0.60 | | 70.02 | | 140 | | -- |
| **Table S4. (continued)** | | | | | | | | | | |
| **Testing strategy** | **Costs (€)^1,2^** | | **QALMs, total population^1^** | | **Transmissions per HIV-infected person** | | **QALMs accrued per transmission^2^** | | **Costs accrued per transmission^1^** | **ICER (€/QALY)^3^** |
| One-time screening | 850 | 194.32 | | 0.61 | | 70.03 | | 160 | | 43,000 |
| ***Overall HIV prevalence = 0.60%*** | | | | | | | | | | |
| *30% decrease in cost of all lines of ART, HIV test cost = €6* | | | | | | | | | | |
| Current practice | 500 | 194.29 | | 0.60 | | 72.93 | | 90 | | -- |
| One-time screening | 540 | 194.31 | | 0.61 | | 72.94 | | 100 | | 21,000 |
| *30% increase in cost of all lines of ART, HIV test cost = €6* | | | | | | | | | | |
| Current practice | 810 | 194.29 | | 0.60 | | 72.93 | | 140 | | -- |
| One-time screening | 870 | 194.31 | | 0.61 | | 72.94 | | 170 | | 33,000 |
| *30% decrease in cost of all lines of ART, HIV test cost = €37* | | | | | | | | | | |
| Current practice | 500 | 194.29 | | 0.60 | | 72.88 | | 90 | | -- |
| One-time screening | 560 | 194.31 | | 0.60 | | 72.89 | | 110 | | 31,000 |
| *30% increase in cost of all lines of ART, HIV test cost = €37* | | | | | | | | | | |
| Current practice | 810 | 194.29 | | 0.60 | | 72.88 | | 140 | | -- |
| **Table S4. (continued)** | | | | | | | | | | |
| **Testing strategy** | **Costs (€)^1,2^** | | **QALMs, total population^1^** | | **Transmissions per HIV-infected person** | | **QALMs accrued per transmission^2^** | | **Costs accrued per transmission^1^** | **ICER (€/QALY)^3^** |
| One-time screening | 900 | 194.31 | | 0.60 | | 72.89 | | 170 | | 43,000 |

1. Costs are rounded to the nearest €10 and discounted at 5% per annum.
2. QALMs are discounted at 5% per annum.
3. Incremental cost-effectiveness with secondary transmission = [(cost per person in strategy 2 + cost accrued per transmission in strategy 2) – (cost per person in strategy 1 + cost accrued per transmission in strategy 1)]/[(QALMs per person in strategy 2 + QALMs accrued per transmission in strategy 2) – (QALMs per person in strategy 1 + QALMs accrued per transmission in strategy 1)]. ICERs are rounded to the nearest €1,000/QALY.

**Figure Legend**

**Figure S1: Conceptual framework for the screening module.** A random number generator assigns each incoming member of the population four, HIV-related event times. A series of “IF/THEN” statements reconciles the sequencing of these events and determines if and when HIV infection takes place, the time and frequency of all HIV Counseling, Testing, and Referral (HIVCTR) activities, and times of HIV screen detection and patient eligibility for therapy. For HIV-infected individuals, this information is conveyed to the Disease Model, which then simulates the progress of HIV disease, clinical care, and all associated costs. For all individuals, the Screening Module records cost and performance data on HIVCTR services.

**Figure S1.**
